# Rapid Increase in frequency of gene copy-number variants during experimental evolution in *Caenorhabditis elegans*

**DOI:** 10.1186/s12864-015-2253-2

**Published:** 2015-12-09

**Authors:** James C. Farslow, Kendra J. Lipinski, Lucille B. Packard, Mark L. Edgley, Jon Taylor, Stephane Flibotte, Donald G. Moerman, Vaishali Katju, Ulfar Bergthorsson

**Affiliations:** Department of Biology, University of New Mexico, Albuquerque, NM 87131 USA; Department of Zoology, University of British Columbia, Vancouver, BC V6T 1Z4 Canada; Present address: Department of Veterinary Integrative Biosciences, Texas A&M University, College Station, TX 77843-4458 USA

## Abstract

**Background:**

Gene copy-number variation (CNVs), which provides the raw material for the evolution of novel genes, is widespread in natural populations. We investigated whether CNVs constitute a common mechanism of genetic change during adaptation in experimental *Caenorhabditis elegans* populations. Outcrossing *C. elegans* populations with low fitness were evolved for >200 generations. The frequencies of CNVs in these populations were analyzed by oligonucleotide array comparative genome hybridization, quantitative PCR, PCR, DNA sequencing across breakpoints, and single-worm PCR.

**Results:**

Multiple duplications and deletions rose to intermediate or high frequencies in independent populations. Several lines of evidence suggest that these changes were adaptive: (i) copy-number changes reached high frequency or were fixed in a short time, (ii) many independent populations harbored CNVs spanning the same genes, and (iii) larger average size of CNVs in adapting populations relative to spontaneous CNVs. The latter is expected if larger CNVs are more likely to encompass genes under selection for a change in gene dosage. Several convergent CNVs originated in populations descended from different low fitness ancestors as well as high fitness controls.

**Conclusions:**

We show that gene copy-number changes are a common class of adaptive genetic change. Due to the high rates of origin of spontaneous duplications and deletions, copy-number changes containing the same genes arose readily in independent populations. Duplications that reached high frequencies in these adapting populations were significantly larger in span. Many convergent CNVs may be general adaptations to laboratory conditions. These results demonstrate the great potential borne by CNVs for evolutionary adaptation.

**Electronic supplementary material:**

The online version of this article (doi:10.1186/s12864-015-2253-2) contains supplementary material, which is available to authorized users.

## Background

Gene and genome duplications are the primary source of new genes and have played a pivotal role in the evolution of genomic and organismal complexity [1–4]. The rates of spontaneous gene duplication and deletion are extraordinarily high and speak to the enormous potential of these structural variants for generating new adaptive variability [5–10]. However, most gene duplicates are eventually lost from populations due to a variety of reasons: genetic drift or natural selection, inherent instability of tandem duplications, and relaxed selection against detrimental mutations [5, 11–14]. Although, gene duplications and deletions contribute significantly to the immense standing genetic variation related to gene copy-number observed in natural populations [15–18], the relative importance of genetic drift versus natural selection in determining their evolutionary fate remains obscure.

Ohno [1] theorized that newly duplicated genes were freed from the constraints of natural selection, implicating a dominant role of genetic drift in their early evolutionary dynamics. Likewise, genetic drift is assumed to be the dominant force in the early evolutionary history of duplicate genes under the DDC (duplication-degeneration-complementation) model [19]. In contrast, natural selection for increased gene expression may represent an important mechanism by which duplicate gene copies are maintained in populations [14]. There is ample evidence for the preservation of multiple gene copies due to selection for increased gene dosage in diverse organisms [20]. For example, adaptation to novel or resource-limited environments in laboratory populations frequently involves segmental duplications [21–24]. Likewise, natural populations harbor duplications that are clearly adaptive under novel environmental regimes [25–29]. In addition, loss-of-function mutations can often be suppressed or compensated for by multiple copies, or increased transcription of another gene in the genome [30–43]. The spontaneous rate of gene deletions is of a similar magnitude as that of duplications [8, 9]. There is evidence that deletions tend to be more detrimental to fitness than duplications [44]. However, gene loss has also been associated with adaptation in diverse systems [45–47].

We have previously established that the spontaneous, genome-wide rate of gene duplication in *C. elegans* is two orders of magnitude higher than the point mutation rate [8]. In this study, we seek to determine if gene copy-number changes are a common class of genetic change during adaptation and what role, if any, natural selection plays in the maintenance and frequency increase of copy-number variants (CNVs henceforth) in experimental populations. Gene copy-number changes were analyzed in experimental lines of *C. elegans* which had been subjected to (i) fitness decline via mutation accumulation, and (ii) subsequent adaptive fitness recovery during population expansion for >200 generations. In addition, control lines maintained at large population sizes without having been subjected to mutation accumulation were also analyzed for copy-number changes. We used an obligately outcrossing strain of *C. elegans* to reduce the effects of genetic hitchhiking [48]. These fitness-recovered populations were subsequently analyzed for copy-number changes to directly test if recovery lines display high rates of duplications and deletions, and to determine the role of these CNVs in adaptive evolution.

## Results

### Fitness decline during mutation accumulation (MA) and subsequent fitness increase following population expansion

**Fig. 1:**
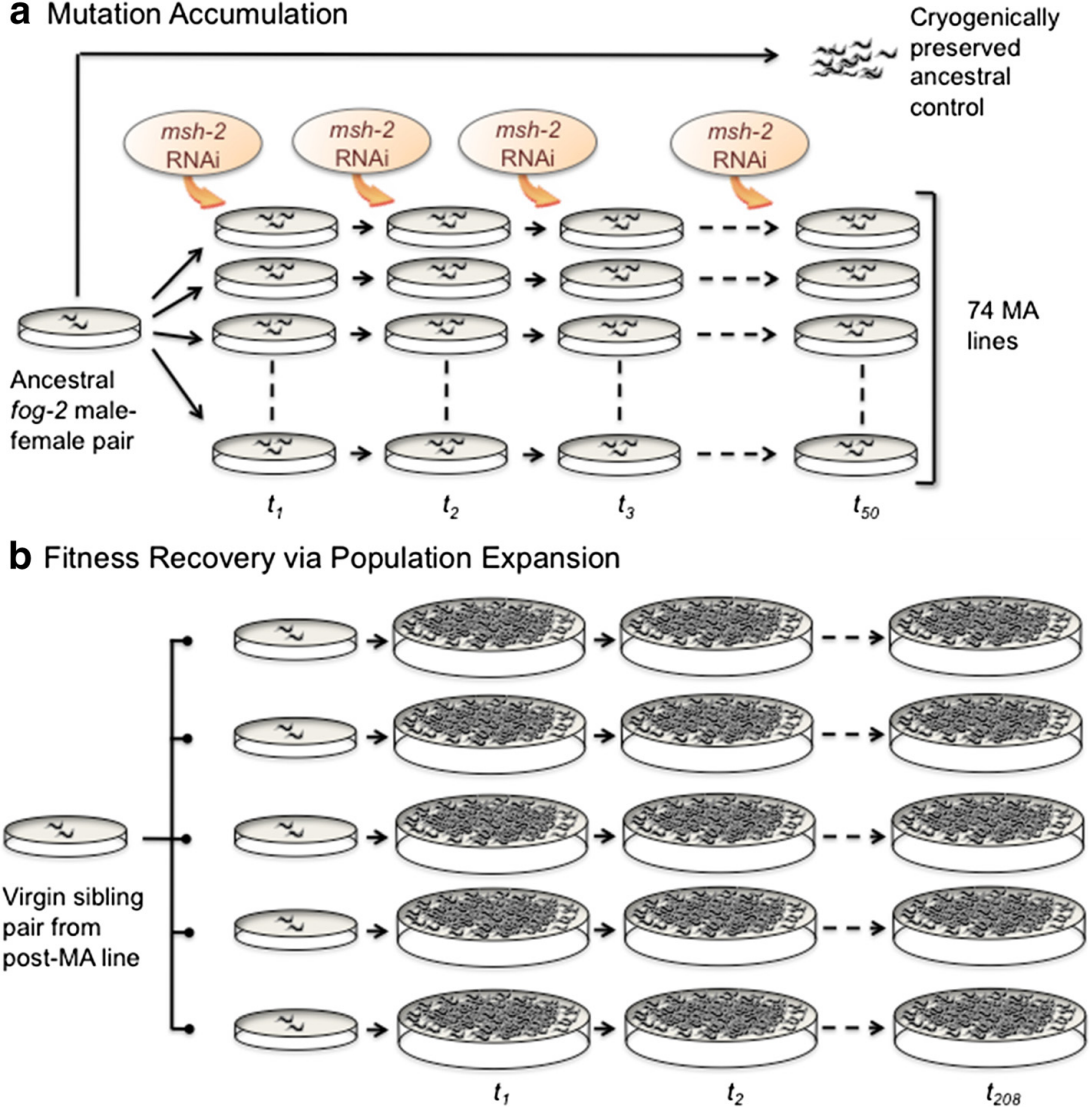
Illustration of *Caenorhabditis elegans* experimental evolution study with mutation accumulation (MA) and adaptive recovery phases. **a** The MA experiment was initiated by establishing 74 lines descended from a single, mated *fog-2* female whose additional descendants were expanded for several generations and frozen as ancestral, pre-MA controls. Each generation, the MA regime comprised (i) population bottlenecks of one random female worm and two male siblings (N_*e*_ = ~2.67) per generation, and (ii) RNAi-mediated knockdown of the mismatch repair gene *msh-2*. The MA experiment with *msh-2* RNAi was terminated at 50 generations and extant MA lines were subjected to 15 additional generations of full-sib mating without *msh-2* RNAi to maximize homozygosity. **b** To enable fitness/adaptive recovery of mutationally degraded lines, five MA lines (MA7, 16, 19, 50 and 66) exhibiting the greatest decline in fitness following the MA regime were expanded into five sublines (A-E) and independently maintained at large population sizes in the absence of *msh-2* RNAi. New generations were established every four days by agar chunk transfers that enabled maintenance of large population sizes across generations. For simplicity, the fitness recovery phase displayed in the figure only depicts population expansion for one MA line and its five descendant sublines, A-E

**Fig. 2:**
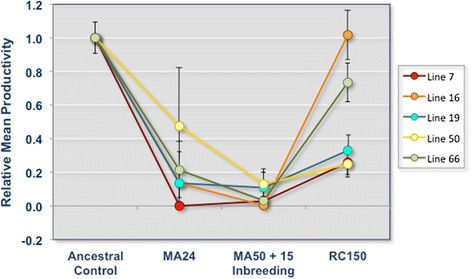
Decline in mean productivity of experimental lines during mutation accumulation with subsequent increase in productivity during population expansion. Fitness (productivity) trajectories of five experimental evolution lines of *C. elegans* during two experimental phases of (a) mutation accumulation, and (b) fitness recovery after population expansion. Two fitness assays were conducted during the mutation accumulation phase of the experiment — (i) following 24 consecutive generations of mutation accumulation with *msh-2* RNAi (MA24), and (ii) 50 consecutive generations of mutation accumulation with *msh-2* RNAi and an additional 15 additional generations of full-sib mating to promote homozygosity (MA50 + 15 Inbreeding). All five experimental lines displayed significant decline in productivity, a fitness-related trait during the MA phase, relative to the ancestral pre-MA control from which all lines were derived. Populations derived from the MA lines exhibited moderate to strong fitness recovery following 150 consecutive generations of maintenance at large population sizes (RC150). Each point for the assay RC150 represents the mean productivity across five independently expanded population and within population replicates (5 populations × 5 replicates per population). The mean productivity of the ancestral pre-mutation accumulation control has been scaled to a value of 1. Errors bars represent one standard error

This experimental evolution study comprised two distinct phases, (i) a mutation accumulation with a *msh-2* knockdown (MA) phase (Fig. 1a), followed by (ii) an adaptive recovery phase in the absence of *msh-2* knockdown (Fig. 1b). Figure 2 displays the fitness trajectories of the five focal experimental lines via three fitness assays spanning both phases of the experiment (MA and population expansion), as measured by the life-history trait productivity. Ancestral pre-MA control lines had a mean productivity value of 464 progeny and were assigned a relative mean productivity value of 1.00. At 24 MA generations, the mean productivity of the five experimental lines ranged from 0.2 – 220 progeny (relative mean productivity of 0.004–47 % compared to the ancestral control, Fig. 2). The mean productivity of the five focal MA lines at the termination of the MA l phase (50 MA generations) was 31 offspring and the individual mean productivity of the five experimental MA lines ranged from 2–60 progeny (relative mean productivity of 0.43–13 % compared to the ancestral control, Fig. 2). ANOVA analyses found a significant variance component for productivity (*F* = 40.1; *p* < 0.0001) between the control and the five MA lines.

Following 150 generations of population expansion, we observed modest to substantial fitness recovery in the experimental lines (Fig. 2). The mean productivity of the 25 adaptive recovery populations (that were descended from five MA lines) ranged from 115–472 progeny, and relative productivity of 0.25–1.02 (25–102 % relative to the ancestor). Populations 16A-E, descended from MA16, exhibited complete fitness recovery to ancestral levels with respect to productivity (average 472 progeny). Populations 66A-E exhibited substantial fitness recovery to 73 % of ancestral levels with respect to productivity (average 341 progeny). Populations 7A-E, 19A-E, and 50A-E, descended from MA7, MA19, and MA50, respectively, had modest increases in productivity, ranging from 25–33 % of ancestral levels (average productivity of 120, 153, and 115, respectively). The mean productivity of the five MA following 50 generations and the 25 recovery populations following ~150 generations was 31 and 274 offspring, respectively. ANOVA analyses found a significant variance component for productivity between the mutation accumulation lines and the recovery populations (*F* = 16.9; *p* < 0.0001).

### CNVs comprise a common class of genetic change during adaptive recovery

oaCGH detected 24 duplication events in 15 of the 25 experimental populations subjected to adaptive recovery following population expansion after mutation accumulation (Table 1). A single duplication event was identified in one of the five *fog-2* control populations (C2), which had been maintained at a large population size without having been subjected to a prior mutation accumulation phase. The duplication spans ranged from 1.6 to 660.8 kb in length, encompassing 1 to 121 protein-coding genes (Table 1 and Additional file 1: Supplemental Data S1). The median duplication span was 191.5 kb and the median number of protein-coding genes per duplication was 38. In addition, there were 18 deletions in 12 of the 25 adaptive recovery populations. An additional seven deletions were observed in the five *fog-2* control populations (one each in C1, C2 and C4; two each in C3, and C5). The length distribution of deletions was markedly different from that of duplications. The deletion spans ranged from 1.1 to 294.6 kb, resulting in the deletion of zero to 38 protein-coding genes (Table 2 and Additional file 2: Supplemental Data S2). The median deletion span was 12.5 kb and the median number of protein-coding genes deleted was one. None of these copy-number changes in the adaptive recovery phase were detected in the MA lines via (i) microarray analysis using the MA lines as the experimental lines and the common ancestor of all MA lines as a reference, (ii) qPCR, and (iii) PCR and sequencing of duplication and deletion breakpoints. Hence, they appear to have occurred and increased in frequency during the population expansion phase associated with adaptive recovery.

**Table 1:** Summary of duplications in experimental *C. elegans* lines following 200 consecutive generations of population expansion

Pop. ID	Chr	Coordinates		Duplication span (bp)	Protein-coding genes	tRNA	piRNA	ncRNA	Pseudo-genes	Transposons	Average copy-number per haploid genome
		Start	Stop								
7B	IV	6,837,055	6,879,497	42,443	5	0	30	10	1	0	2.19
7B	V	19,505,848	20,101,145	595,298	95	0	32	0	43	6	1.62
7D	IV	505,050	701,113	196,064	38	3	0	15	1	1	1.72
16B*	V	19,295,123	19,839,705	544,583	110	1	2	19	52	1	1.19
16C	IV	9,054,304	9,457,751	403,448	89	0	30	13	8	3	1.23
16C	V	800,408	1,103,333	302,926	57	1	2	12	2	3	1.59
16D	II	6,248,049	6,406,772	158,724	48	0	2	19	1	0	1.53
16D	V	19,746,828	19,885,746	138,919	26	0	2	10	8	1	1.46
16E*	V	19,295,580	19,840,162	544,583	110	1	2	19	52	1	2.08
19C	V	7,637,941	7,641,911	3,971	3	0	1	0	0	0	1.50
19C	II	14,037,517	14,039,164	1,648	1	0	0	0	0	0	1.74
19E	X	813,802	821,373	7,572	2	0	0	0	0	0	1.68
19E	X	829,580	835,392	5,812	2	0	0	0	0	0	1.56
50A*	V	19,780,484	19,972,052	191,569	30	0	2	7	8	4	1.50
50A	X	8,624,771	9,024,484	399,714	64	29	6	59	0	3	1.34
50B	V	19,781,064	19,972,507	191,444	30	0	2	7	8	4	1.66
50C	V	19,659,829	19,976,506	316,680	58	1	2	16	20	5	1.39
50D	IV	560,240	1,024,886	464,647	84	4	1	25	2	2	1.19
50D	V	18,703,541	18,723,878	20,338	4	0	0	2	5	0	1.39
50D	V	19,780,935	19,966,260	185,326	30	0	2	8	8	2	1.78
50E	II	6,312,598	6,444,674	132,077	32	0	1	30	1	1	1.34
50E	V	19,780,952	19,966,162	185,211	30	0	4	9	8	2	1.69
66C	V	19,393,526	20,054,330	660,805	121	1	2	26	52	6	1.51
66E*	V	19,295,300	19,839,882	544,583	111	1	2	29	50	4	1.33
C2*	V	19,295,101	19,839,683	544,583	111	1	2	29	50	4	1.64

Pop. ID refers to the experimental population number. Columns 2, 3 and 4 display the chromosomal location of the CNV and the chromosomal coordinates based on WormBase version WS243. Column 5 provides estimates of the span (bp) of the duplication. Columns 6–11 represent the number of protein-coding genes, tRNAs, piRNAs, ncRNAs, pseudogenes and transposases, respectively, that are encompassed by the duplication event. Column 12 represents the copy-number of the duplicated region in the population following 180–212 generations of population expansion based on oaCGH results. Chromosomal coordinates of duplications are predicted based on oaCGH probes in all cases except for events marked by a * wherein exact duplication breakpoints were determined via direct sequencing

**Table 2:** Summary of deletions in experimental *C. elegans* lines following 200 consecutive generations of population expansion

Pop ID	Chr.	Coordinates		Delation span (bp)	Protein-coding genes	tRNA/rRNA	piRNA	ncRNA	Pseudo-genes	Transposons	Average copy-number per haploid genome
		Start	Stop								
16A*	X	817,573	830,086	12,514	1	0	0	0	0	0	0.05
16D*	V	7,663,133	7,687,447	24,315	7	0	0	0	0	0	0.05
19A*	X	800,773	827,100	26,328	5	0	1	0	0	0	0.47
19C	V	7,642,395	7,682,740	40,346	10	0	0	1	0	0	0.23
19E	X	821,499	829,454	7,956	1	0	0	0	0	0	0.19
50B	V	7,650,284	7,693,435	43,152	12	0	0	0	1	0	0.76
50C	V	7,647,125	7,696,096	48,972	14	0	0	0	1	0	0.71
50C	X	1,029	273,082	272,054	35	0	0	14	18	5	0.85
50D*	V	7,653,667	7,680,465	26,799	6	0	0	0	0	0	0.18
50D	X	1,029	295,671	294,643	38	0	0	15	20	6	0.81
50E*	V	7,652,044	7,682,914	30,871	8	0	0	0	0	0	0.47
66B	V	15,258,727	15,326,180	67,454	26	0	1	2	5	1	0.62
66B*	X	9,983,441	9,999,107	15,667	2	0	0	1	0	0	0.04
66D	V	18,665,661	18,670,354	4,694	1	0	0	0	0	0	0.54
66D	V	18,701,820	18,725,404	23,585	3	0	0	3	5	0	0.39
66D	X	961,361	963,014	1,654	1	0	0	0	0	0	0.09
66D	X	7,528,608	7,529,729	1,122	1	0	0	0	0	0	0.07
66E	X	7,528,608	7,529,729	1,122	1	0	0	0	0	0	0.06
C1	I	15,060,622	15,071,438	10,817	0	4	0	4	1	0	0.75
C2	I	15,060,388	15,071,427	11,040	0	4	0	4	1	0	0.66
C3	II	14,034,460	14,039,471	5,012	1	0	0	0	0	0	0.45
C3*	X	7,527,813	7,529,236	1,424	1	0	0	0	0	0	0.05
C4	I	15,060,388	15,071,427	11,040	0	4	0	4	1	0	0.60
C5	I	15,061,973	15,071,438	9,466	0	4	0	3	0	0	0.79
C5	X	823,167	827,286	4,120	1	0	0	0	0	0	0.38

Pop. ID refers to the experimental population number. Columns 2, 3 and 4 display the chromosomal location of the CNV and the chromosomal coordinates based on WormBase version WS243. Column 5 provides estimates of the span (bp) of the deletion. Columns 6–11 represent the number of protein-coding genes, tRNAs/rRNAs, piRNAs, ncRNAs, pseudogenes and transposases, respectively, that are encompassed by the deletion event. Column 12 represents the copy-number of the deleted region in the population following 180–212 generations of population expansion based on oaCGH results. Chromosomal coordinates of deletion are predicted based on oaCGH probes in all cases except for events marked by a * wherein exact deletion breakpoints were determined via direct sequencing

### Duplications and deletions during adaptive recovery are significantly larger than those arising under mutation accumulation conditions

**Fig. 3:**
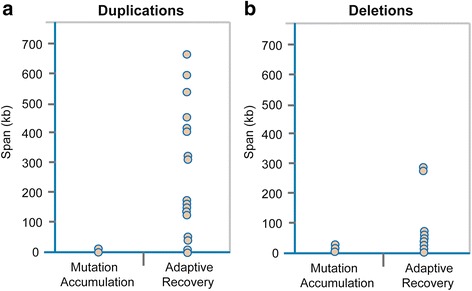
Comparison of duplication and deletion spans in adaptive recovery versus spontaneous mutation accumulation (MA) lines. **a** The span of 24 independent duplication events in the adaptive recovery populations compared to the duplication span of 14 spontaneous duplications during MA [8]. The span of duplications during adaptive recovery is significantly larger than duplications detected under spontaneous MA conditions (*p* < 0.0001). **b** The span of 18 deletion events in the adaptive recovery populations compared to the deletion span of 11 spontaneous deletions during MA [8]. The deletion span for 18 deletion events in the adaptive recovery populations was significantly greater than the span of spontaneous deletions during MA (*p* = 0.032)

We further compared the size of CNVs originating in the adaptive recovery populations to spontaneously-occurring CNVs previously investigated in *C. elegans* lines comprising a long-term MA experiment with extreme bottlenecks of *N*_*e*_ = 1 [8]. The duplication span in our adaptive recovery populations is significantly greater than that of previously determined spontaneous duplications under mutation accumulation conditions [8] (Wilcoxon two-sample test, *Z* = −3.85, *p* < 0.0001, Fig. 3a). Duplications in populations subjected to adaptive recovery had a median duplication span of 191.5 kb versus a median span of 7.2 kb in spontaneous mutation accumulation populations [8] under the influence of genetic drift. Similarly, we detected significantly larger deletion spans in the adaptive recovery populations compared to spontaneous deletions occurring under mutation accumulation conditions (Wilcoxon two-sample test, *Z* = −2.4, *p* = 0.016, Fig. 3b). The median spans of deletions in our adaptive recovery and mutation accumulation populations [8] were 12.5 and 3.5 kb, respectively.

### Gradual increase in the frequencies of CNVs during the adaptive recovery phase

Based on the oaCGH arrays, the average population wide copy-number of the 24 duplications ranged from 1.19 to 2.19 copies per haploid genome (Table 1). Assuming that individuals harboring duplications only contain one additional copy of the duplicated segment, the frequency of individual duplications in the populations range from 0.19 to 1 (or fixation). The average copy-number for the deleted segments ranged from 0.81 to 0.04, suggesting that the frequency of these deletions in the populations range from 0.19 to 0.96.

**Fig. 4:**
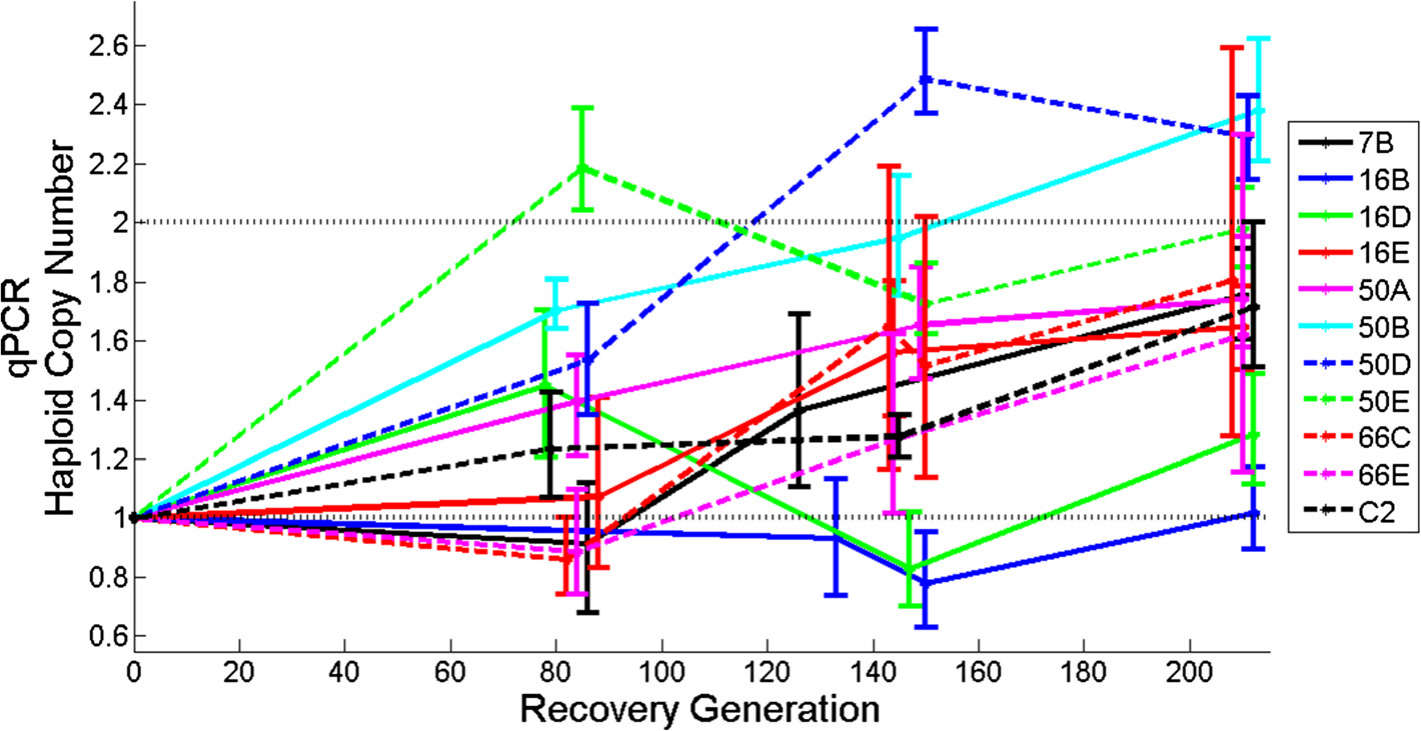
Increase in the frequency of parallel duplication events in 11 independent populations containing an overlapping region on Chromosome V. The average copy-number per haploid genome was calculated from qPCR results and is indicated on the vertical axis. The vertical lines indicate the 95 % bootstrap confidence intervals. The generation from which the copy-number was estimated is indicated on the horizontal axis

**Fig. 5:**
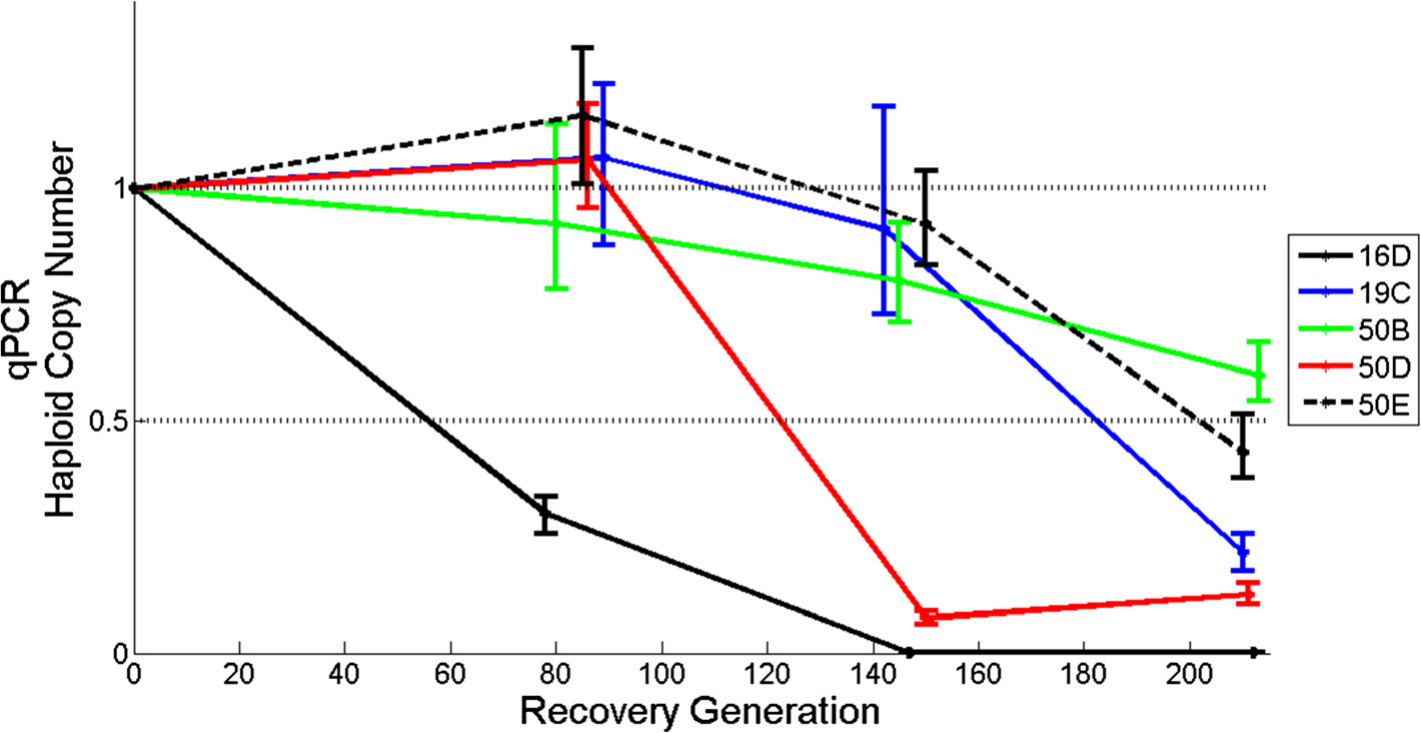
Copy-number decreases due to parallel deletion events in five adaptive recovery populations containing an overlapping region on Chromosome V. The average copy-number per haploid genome was calculated from qPCR results and is indicated on the vertical axis. The vertical lines indicate the 95 % bootstrap confidence intervals. The number of recovery generations is indicated on the horizontal axis. The deletions have reached fixation when the average copy-number has reached 0

In light of the oaCGH results following >200 recovery generations, qPCR was used to analyze the frequencies of duplications and deletions following approximately 80, 140 and, 208 recovery generations. In the majority of the populations, duplications and deletions that had reached high frequencies by generations 180–212 were found in intermediate frequencies at approximately 80 and 140 generations, providing evidence of a gradual increase in the frequencies of individual CNVs with time (Figs. 4 and 5; Additional file 3: Figure S1, Additional file 4: Figure S2, Additional file 5: Figure S3, Additional file 6: Figure S4, Additional file 7: Figure S5, Additional file 8: Figure S6, Additional file 9: Figure S7 and Additional file 10: Figure S8). Based on the oaCGH results in Table 1, duplications in two populations had reached fixation by recovery generation 208 (7B:ChrIV, and 16E:ChrV). However, based on the qPCR results, three additional duplications appear to have reached fixation in their respective populations (19E:ChrX, 50B:ChrV, and 50D:ChrV) (Fig. 4 and Additional file 6: Figure S4). The pattern of increase in the frequency of CNVs is particularly striking in the case of several deletions (Table 2, Fig. 5 and Additional file 7: Figure S5, Additional file 8: Figure S6, Additional file 9: Figure S7 and Additional file 10: Figure S8). The oaCGH results suggested that six deletions reached high frequency and that the deleted segment is only in 4–9 % frequency in these populations (Table 2). Moreover, the qPCR results for these CNVs suggest that five deletions were already fixed by recovery generations 140–160 in these populations (Fig. 5, Additional file 8: Figure S6, Additional file 9: Figure S7 and Additional file 10: Figure S8 corresponding to 16A:ChrX, 16D:ChrV, 2 deletions in 66D:ChrX, and 66E:ChrX) and one additional deletion (66B:ChrX; Additional file 10: Figure S8) had reached fixation by recovery generation 208. In general, there was a good correlation between the oaCGH and qPCR estimates of the frequency of copy-number changes (duplications and deletions) in the populations at recovery generation 208 (*r* = 0.95, *p* < 0.001).

### Duplication breakpoints in independent populations occur at unique sites within the same repetitive sequences

**Fig. 6:**
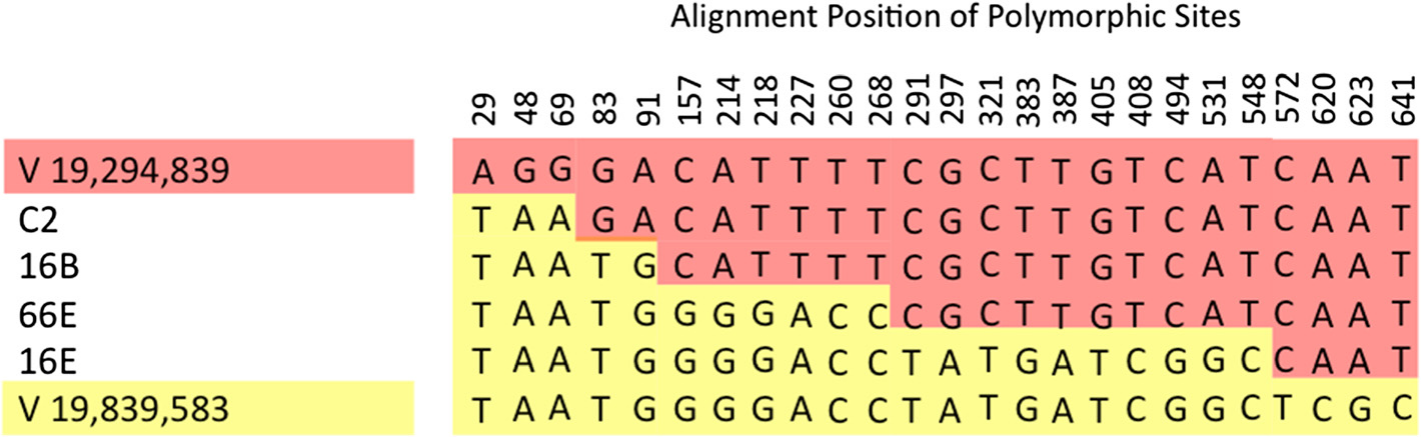
Breakpoints of the four common duplications on chromosome V compared to their flanking repeats. Four independent populations contain a duplication of a region between positions 19,294,839 and 19,838,583 on chromosome V. These duplications are the product of unequal crossing-over between two 1,031 bp repeats that are 96 % identical and flank the duplication. The figure shows polymorphic sites between the two repeats, and the nucleotides flanking the breakpoints of the four duplications. The sequences of the upstream and downstream repeats are displayed on the topmost (*orange*) and lowermost (*yellow*) rows, respectively. The sequence of the new repeat in the center of the tandem duplication is shown for strains 16B, 66E, 16E, and C2, and the correspondence to the original flanking repeats is indicated by color. The duplication breakpoint is inferred to be between the sequence that corresponds to the downstream repeat *(yellow*) and the upstream repeat (*orange*)

Our attempts to precisely map the duplication and deletion breakpoints with PCR and DNA sequencing yielded mixed results. We were able to sequence five duplication breakpoints from the set of 24 duplications in Table 1. In addition, we generated breakpoint sequences for seven deletion events in Table 2. Four duplication breakpoints on chromosome V, in populations 16B, 16E, 66E and control population C2, are located within the same 1031 bp repeats flanking the duplications and appear to be the result of unequal crossing-over. The sequence identity between the two repeats is 96 % and the point of unequal crossing-over within the repeats is different in all four cases, confirming that these were independent events (Fig. 6). The seven deletions with sequenced breakpoints are 16A:ChrX, 16D:ChrV, 19A:ChrX, 50D:ChrV, 50E:ChrV, 66B:ChrX, and C3:ChrX (Table 2). These sequenced deletions do not appear to be associated with repeat motifs.

### Extensive parallelism in copy-number changes of certain CNVs

**Fig. 7:**
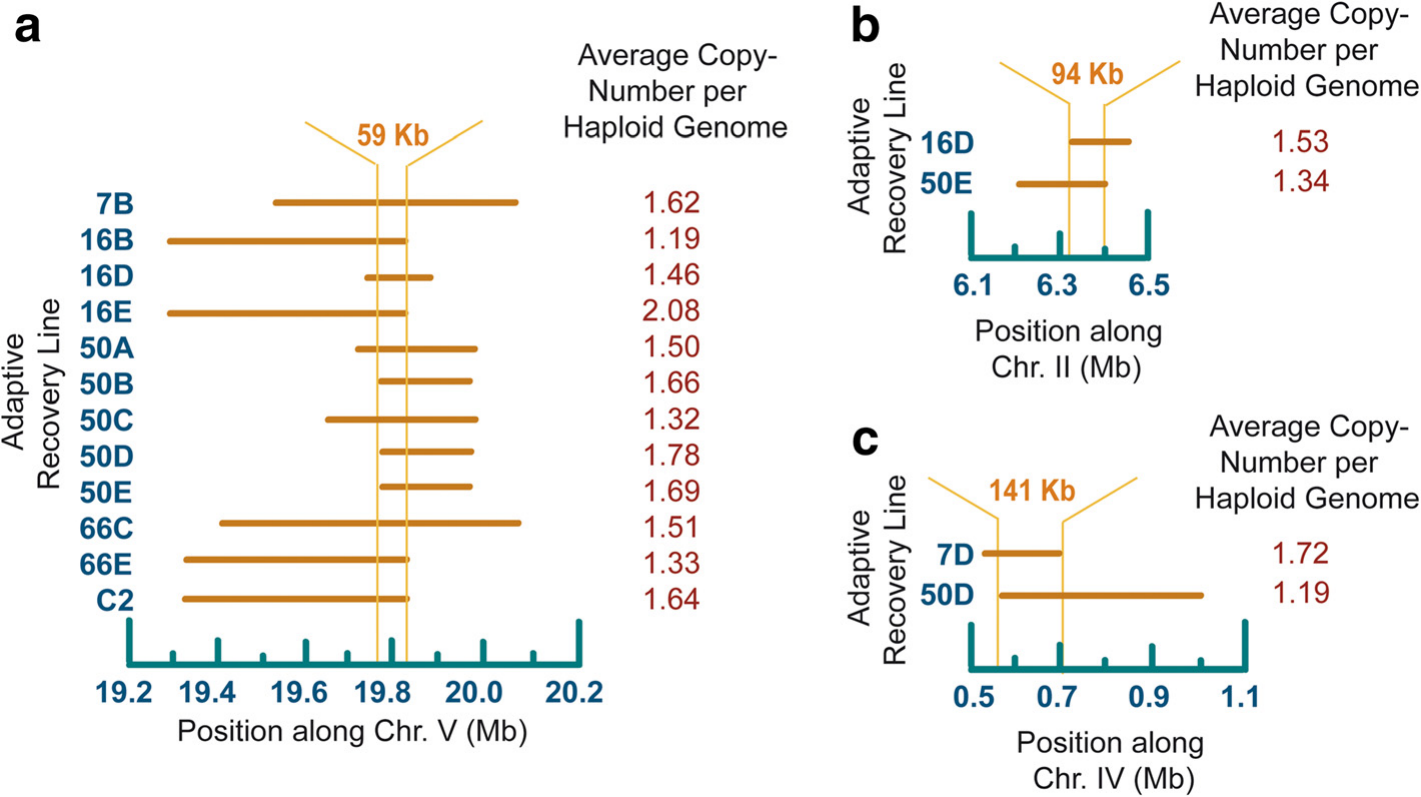
Location and span of convergent duplication events. The populations are indicated to the left, the chromosomal position is shown on the horizontal axis and the average haploid copy-number based on the oaCGH results from generation 208 is indicated on the right. The horizontal bars designate the regions that are duplicated in each of these populations. The vertical orange lines indicate the boundaries of the shared segment among these duplications. **a** Overlapping duplications on chromosome V during the adaptive recovery phase of the experiment. The 59 kb region shared among all 12 populations is delineated by the vertical lines that run through the horizontal bars. **b** Overlapping duplications on chromosome II during the adaptive recovery phase of the experiment. The 94 kb region shared among the two populations is delineated by the vertical lines that run through the horizontal bars. **c** Overlapping duplications on chromosome IV during the adaptive recovery phase of the experiment. The 141 kb region shared among the two populations is delineated by the vertical lines that run through the horizontal bars

Twelve duplications in 11 independent recovery populations and one control population span an overlapping region on chromosome V which extends up to ~59 kb and contains 11 protein-coding genes (Fig. 7a and Additional file 11: Supplemental Data S3). The range of duplication spans encompassing this overlapping region in the 12 populations range from ~139–661 kb. Gene Ontology (GO) annotations report the function of four of these 11 duplicated ORFs (*srt-45*, M162.7, Y116F11B.2, and Y116F11B.17) as unclassified with respect to biological process, cellular component and molecular function. Four of the 11 duplicated ORFs have their molecular function defined as protein-binding (*fbxa-118*, and *fbxa-194*) or carbohydrate-binding (*clec-258*, and *clec-259*). Duplicated gene *daf-28* is probably the best-characterized locus within this shared region on chromosome V. It encodes a beta-type insulin and inhibits dauer formation [49] and influences adult life-span, two potentially important life-history traits that could be under selection during the adaptive recovery regime of the experiment. *pcp-4* exhibits serine-type peptidase activity and is involved in proteolysis whereas *srw-38* codes for a protein product that serves as an integral component of membranes.

The convergent duplications on chromosome II (populations 16D and 50E), (Fig. 7b and Additional file 11: Supplemental Data S3) and chromosome IV (populations 7D and 50D), (Fig. 7c and Additional file 11: Supplemental Data S3) encompass larger overlapping regions (94 kb and 141 kb, respectively), and have lower average copy-numbers relative to the convergent duplications on chromosome V (Fig. 7a). The convergent or overlapping duplications on Chromosome II are found in two populations and span 26 protein-coding ORFs of which 11 are unclassified with respect to biological process, cellular component and molecular function. For the remaining 15 ORFs, we note that ten ORFs (C32D5.3, *sma-6*, *set-4*, C32D5.8, *lgg-1*, C32D5.10, C32D5.12, *ani-2*, *lin-23*, and F58F12.1) have biological processes related to important life-history traits involving some combination of reproduction, dauer development, embryo development, determination of adult lifespan and oogenesis. The convergent duplications on chromosome IV occur in two populations and span 30 protein-coding ORFs of which 18 are unclassified with respect to biological process, cellular component and molecular function. Of the remaining 12 ORFs, six ORFs (*efn-4*, *gex-2*, F56A11.6, *rpl-15*, K11H12.3, and *cutl-28*) have biological processes related to the very same life-history traits observed for the overlapping duplication on chromosome II.

**Fig. 8:**
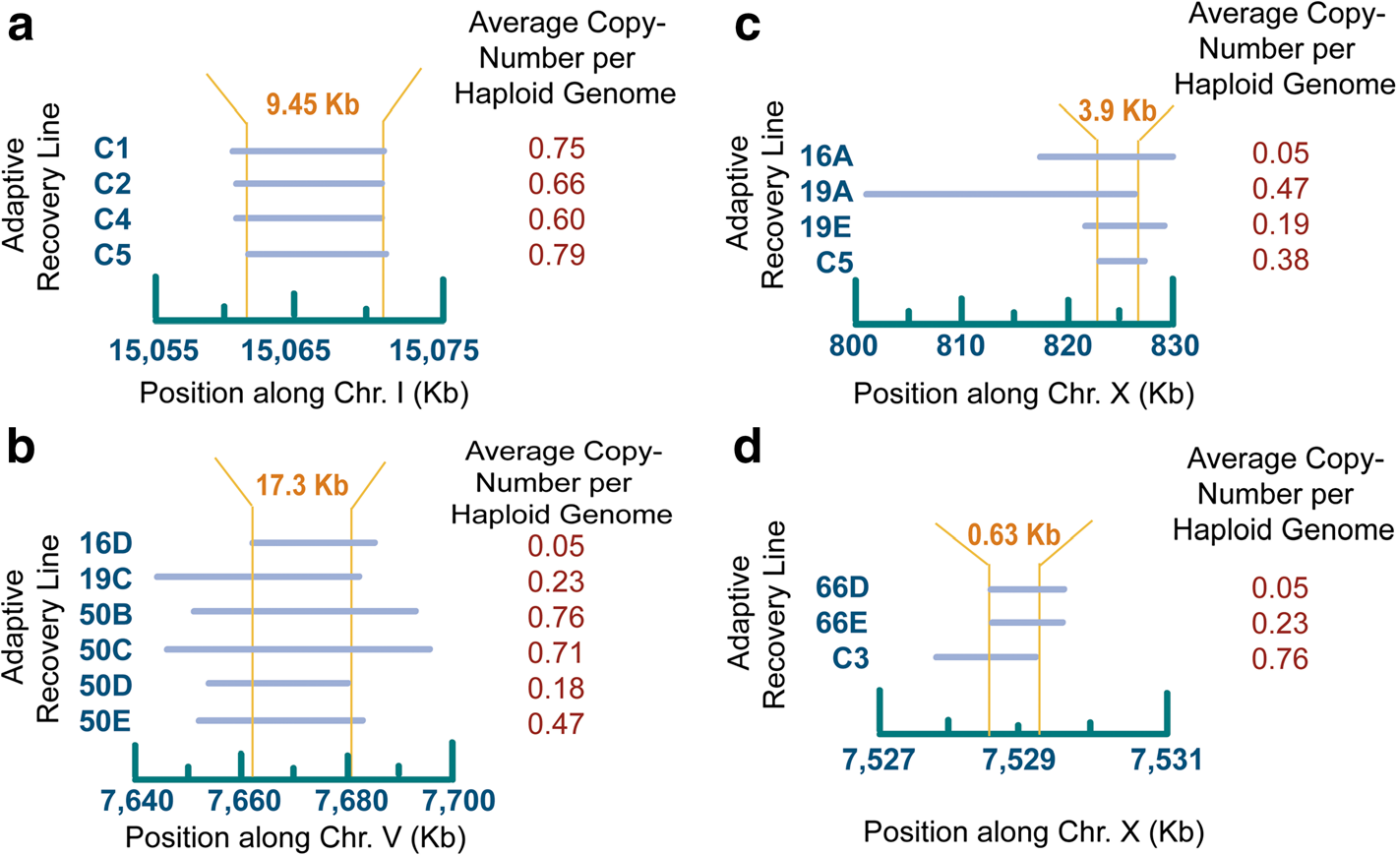
Location and span of convergent deletion events. The populations are indicated to the left, the chromosomal position is shown on the horizontal axis and the average haploid copy-number based on the oaCGH results from generation 208 is indicated on the right. The horizontal bars designate the regions that are deleted in each of these populations. The vertical orange lines indicate the boundaries of the shared segment among these deletions. **a** Overlapping deletion on chromosome I during the adaptive recovery phase of the experiment. The ~9.5 kb region shared among four control populations (C1, C2, C4 and C5) is delineated by the vertical lines that run through the horizontal bars. **b** Overlapping deletion on chromosome V during the adaptive recovery phase of the experiment. The 17.3 kb region shared among the six adaptive recovery populations is delineated by the vertical lines that run through the horizontal bars. **c** Overlapping deletions on chromosome X during the adaptive recovery phase of the experiment. The 3.9 kb region shared among three adaptive recovery and one control population(s) is delineated by the vertical lines that run through the horizontal bars. **d** Overlapping deletions on chromosome X during the adaptive recovery phase of the experiment. The 0.6 kb region shared among the two adaptive recovery and one control population(s) is delineated by the vertical lines that run through the horizontal bars

Additionally, we also observed five convergent deletions that spanned overlapping regions in independent populations. Cumulatively, these five convergent deletions comprise 19 independent deletion events observed in 11 adaptive recovery populations and all five control populations. One convergent deletion in four control populations of the adaptive recovery phase (C1, C2, C4 and C5) spanned ~9.5 kb and resulted from a copy-number loss in four rDNA genes at the end of chromosome I (F31C3.7, F31C3.11, F31C3.9, and F31C3.8) (Fig. 8a and Additional file 11: Supplemental Data S3). Our qPCR results suggest that the *fog-2* strain, ancestral to all of the populations in these experiments, possesses 86 copies of this repeat. In these four control populations, the number of rDNA repeats has been reduced by 21–40 % (Table 2).

A second convergent deletion event was detected in six adaptive recovery populations (16D where it appears to have reached fixation, 19C, 50B, 50C, 50D, and 50E) and led to the loss of an overlapping 17,333 bp region on chromosome V encompassing four protein-coding ORFs (Figs. 5 and 8b and Additional file 11: Supplemental Data S3). Three of these ORFs are unclassified with respect to GO annotations. The last ORF, *Cyp-33A1* (C12D5.70), was partially deleted and is classified as a heme- and iron-ion binding protein involved in the oxidation-reduction process.

The third convergent deletion event occurred in three adaptive recovery populations (16A, 19A, 19E) and one control population (C5). This deletion entailed the loss of an overlapping 3934 bp region partially encompassing a single protein-coding gene, *daf-3* (F25E2.5) on chromosome X (Fig. 8c, Additional file 2: Supplemental Data S2 and Additional file 11: Supplemental Data S3). *daf-3* is classified as an enhancer sequence-specific DNA-binding protein involved in dauer larval development among its biological processes.

The fourth convergent deletion event occurred in three populations (66D, 66E, C3) resulting in the loss of an overlapping 629 bp region partially encompassing a single protein-coding gene, *ceh-14* (F46C8.5) on chromosome X (Fig. 8d and Additional file 11: Supplemental Data S3). *ceh-14* is classified as a DNA- and protein-binding protein involved in the regulation of transcription and thermosensory behavior, with *ceh-14* mutants exhibiting lack of thermotaxis. In all cases, the deletion appears to have reached fixation within the populations. Although two of these deletions occurred in populations undergoing adaptive recovery following MA, one occurred in a control population that had not been subjected to MA and adaptive recovery. Interestingly, a lone deletion event in another gene on the X chromosome implicated in thermotaxis [50], *ncs-1*, also reached fixation in strain 66D (Table 2).

Lastly, a fifth convergent deletion event occurred in two adaptive recovery populations, 50C and 50D. This deletion resulting in the loss of one end of the X chromosome reached a significant frequency in both populations. The deletion span in 50D was approximately 22 kb larger than the deletion in 50C. The average haploid copy- number of this segment was 0.85 and 0.81 in 50C and 50D, respectively, which translates into 15 % and 19 % of the X chromosomes bearing this segmental deletion in populations 50C and 50D, respectively. The overlapping 272 kb region in these two deletions contains 35 protein-coding genes (Additional file 11: Supplemental Data S3). 20 of these 35 ORFs are unclassified with respect to GO annotations. For the remaining 15 ORFs, six ORFs (Y73B3A.18, Y73B3A.3, *elk-2*, *cad-6*, Y73B3A.10 and *set-33*) have biological processes related to important life-history and developmental traits involving some combination of reproduction, embryo development ending in birth or egg hatching, nematode larval development, hermaphrodite genitalia development and negative regulation of vulval development.

### Single-worm PCR suggests simple duplications rather than higher-level amplifications

Independent estimates of CNV frequencies via single-worm PCR of CNV breakpoints confirmed the gradual increase of CNVs and are strongly correlated with the copy-number estimates from qPCR (*r* = 0.9; Table 3). There was one instance where the single-worm PCR results deviated significantly from the qPCR results, in line 16B following 212 generations of adaptive recovery. Both the qPCR and oaCGH data suggest that the duplication was present in low frequency in generation 212. In contrast, single-worm PCR estimated the duplication to exist at an intermediate frequency of 0.48 in the population. It is possible that some of the copy-number increases in these populations are due to a higher level of amplification (more than two copies per chromosome) than a single duplication. If the copy-number is frequently > two per haploid genome, we expect that the copy-number calculated from qPCR would systematically exceed the estimates from single-worm PCR. However, this is not the case, and the generally good agreement between the different methods suggests that higher-level amplification is not widespread for the three duplications with single-worm PCR estimates.

**Table 3:** Frequencies of CNVs in experimental *C. elegans* lines at different time intervals of population expansion using single-worm PCR

Pop ID. generation	Type of rearrangements	Individuals sampled	Number positive for rearrangement	Expectec Hardy-Weinberg frequency	Average copy-number	qPCR	oaCGH
16B.80	Duplication	19	0	0.00	1.00	-	-
16B.140		47	0	0.00	1.00	0.86	-
16B.200		43	28	0.41	1.41	1.02	1.19
16E.80	Duplication	30	1	0.02	1.02	1.08	-
16E.140		30	25	0.59	1.59	1.58	-
16E.200		18	18	1.00	2.00	2.39	2.08
66E.140	Duplication	27	10	0.20	1.20	1.23	-
66E.200		28	22	0.54	1.54	1.67	1.33
16A.140	Deletion	30	30	1.00	0.00	0.00	-
16A.200		27	27	1.00	0.00	0.00	0.05
16D.80	Deletion	18	15	0.64	0.36	0.22	-
16D.140		18	28	1.00	0.00	0.000232	-
16D.200		29	29	1.00	0.00	0.000738	0.05
66B.80	Deletion	32	0	0.00	1.00	0.73	-
66B.140		15	9	0.40	0.60	0.40	-
66B.200		28	28	1.00	0.00	0.0000269	0.04

Pop ID.Generation refers to the experimental population number and the number of generations of population expansion when sampled. Column 5 displays the frequency of individuals with the rearrangement assuming Hardy-Weinberg equilibrium. Columns 6–8 show the average copy-number per haploid genome for three methods, namely single-worm PCR, qPCR and oaCGH

## Discussion

In the last decade, analysis of gene copy-number variation has shown that CNVs are surprisingly widespread in natural populations. Like other classes of mutations, these variants can be beneficial, neutral or deleterious. However, gene copy-number increases are unique among mutations in that they can facilitate the evolution of novel genes. The population dynamics of gene copy-number variation in populations are therefore important for understanding both the adaptation and evolution of novel genes. In this study, we investigated whether gene copy-number changes (duplications and deletions) constituted a common form of genetic change during the adaptation of low-fitness experimental populations of *C. elegans*.

Several lines of evidence suggest that the high frequency of copy-number changes in the adaptive recovery and control populations are primarily due to natural selection. Both deletions and duplications increased in frequency with time, and some rearrangements had already reached fixation by 145 generations of population expansion. The theoretical expectation for the average number of generations until fixation of a neutral mutation under conditions of genetic drift is *4N*_*e*_ generations [51]. Assuming a lower-bound conservative estimate of *N*_*e*_ = 1000 individuals in the adaptive recovery populations each generation, neutral CNVs in our experimental populations would take, on average, more than 4000 generations to reach fixation. Five duplications and eight deletions in our adaptive recovery and control populations originated and reached fixation within only 212 generations. Moreover, the majority of other CNVs that had not yet reached fixation by the end of the recovery phase still exhibited a steady increase in population frequency with time. Furthermore, both duplications and deletions contained striking examples of parallelism or convergent evolution. Certain duplications and deletions contained overlapping regions, *i.e*. the same region was duplicated or deleted independently in different populations (Figs. 7 and 8).

Duplications of parts of chromosome V contained the same 59 kb region in eleven independent adaptive recovery populations and one control population (Fig. 7a). If these duplications had been experiencing selection for higher dosage, one or more of these genes could be under selection in all 12 strains. One of the best-characterized genes within this overlapping duplication was *daf-28*, a pleiotropic gene influencing several life-history traits such as adult lifespan and suppression of dauer formation. For instance, if a copy-number increase entails greater *daf-28* expression, the incidence of dauer formation may be further suppressed. In another example of convergence, *daf-3* is deleted in three independent adaptive recovery populations and one control population (Fig. 8c). *daf-3* promotes dauer formation and the deletion is expected to suppress dauer. Hence, we have convergent duplications and deletions in 16 independent populations that are expected to reduce the incidence of dauer formation. We hypothesize that both the duplication of *daf-28* and deletion of *daf-3* may be adaptations to a predictable and frequent availability of a food source, in this case a fresh lawn of *Escherichia coli*. Other examples of convergence in these populations include the partial deletion of a gene, *ceh-14,* in three populations as detected by oaCGH (Fig. 8d). The *ceh-14* gene contributes to thermosensing and thermotaxis in *C. elegans* [52]. Another gene implicated in thermotaxis, *ncs-1*, is also deleted in strain 66D [50].

This form of parallel evolution is best explained by selection for increased gene dosage in the case of duplications [16, 22, 25, 27], and selection against a gene in the case of the deletions [45–47]. Parallel molecular evolution is frequently observed in experimental population studies, particularly in microbial systems [53–56]. In large microbial populations, the chance that the same beneficial mutation will occur in independently-evolving lineages is reasonably high. Compensatory evolution experiments with hermaphroditic *C. elegans* populations have also found parallel nucleotide substitutions at two sites in two independent populations [57]. The high frequency of parallel gene copy-number changes following the population expansion phase in this study is likely due to the high rates of spontaneous copy-number mutations in concert with natural selection [7–9]. Because spontaneous gene duplications and deletions originate at rates that are orders of magnitude higher than point mutations, the probability that copy-number changes in the same genes occur in independent populations is much greater than the same point mutation occurring in independent populations. Furthermore, higher mutation rates improve the probability that new variants increase in frequency or reach fixation [8, 58].

There is a striking difference in the size distribution of spontaneous duplications and deletions detected in MA studies and their size distribution in these populations undergoing adaptive recovery. In a preceding *C. elegans* spontaneous mutation accumulation experiment with minimal influence of natural selection, the spontaneous duplications ranged from 1–30 kb in length, with a median duplication span of 2 kb [8]. In this study of duplications and deletions in adapting *C. elegans* populations following an experimental phase of fitness decline, the size range of duplications originating in the adaptive recovery phase with population expansion was 1.6–661 kb with a median duplication span of 191.5 kb. A similar trend was observed in the case of deletions originating in the adaptive recovery phase. The spontaneous deletions originating during the mutation accumulation experiment ranged from 0.2–32 kb in length, with a median deletion span of 3.5 kb [8]. During the adaptive recovery phase in this study, the size range of deletions was 1.1–295 kb and the median deletion span was ~12.5 kb. Admittedly, we are comparing the size distributions of CNVs in two different strains, the selfing laboratory strain N2 [8] and the obligately outcrossing loss-of-function *fog-2* strain in this study. The large difference in the size distribution can be explained by selection for gene dosage in the recovery populations. The larger the CNV span, the greater the chance that a gene (or several genes) under selection for altered gene dosage will be contained within the duplication or deletion. This may be a general phenomenon and we predict that recent copy-number variants that are being maintained in natural populations are, on average, larger than the average spontaneous duplication or deletion.

It is possible that sex-biased transmission of copy-number changes contribute to differences in the span of duplications and deletions between mutation accumulation experiments in self-fertilizing *C. elegans* and the outcrossing populations in this study. There is evidence that smaller chromosomes tend to segregate with the X chromosome in *C. elegans* [59]. This sex-biased transmission would not influence the distribution of duplication and deletion spans in outcrossing populations such as the *fog-2* mutants in the experiments described here. The transmission bias could introduce a downward bias in duplication span and an upward bias in deletion span in selfing *C. elegans*. Hence, we would expect to see larger duplications and smaller deletions in outcrossing populations relative to the mutation accumulation lines. However, this bias may be negligible in MA experiments with *C. elegans* hermaphrodites because gametes lacking the X chromosome are produced infrequently (<0.1 %) and the opportunities for sex-biased transmission to favor shorter chromosomes in gametes containing the X chromosome would be very limited. Moreover, the results presented here show that both duplications and deletions are larger in the adaptive recovery populations than in mutation accumulation lines, which is not predicted by the transmission bias hypothesis. Additionally, the appearance and increase in the frequency of gene duplications and deletions in large adaptive recovery populations is unlikely to be a direct consequence of the *msh-2* treatment during mutation accumulation. First, following the completion of the MA phase, the experimental lines were inbred for 15 additional generations in the absence of *msh-2* knockdown via RNAi, so it is unlikely that there are any residual effects of the RNAi treatment *per se*. Moreover, all the copy-number changes reported here were not detected in the post-MA ancestor and appear to have arisen during the adaptive recovery phase of the experiment.

Four of 12 populations that contained a large overlapping duplication on chromosome V (Fig. 7a) possessed duplication breakpoints in the same 1 kb repeats (Fig. 6). These repeats appear to be duplication hot-spots. However, this type of duplication was not detected in our previous study of the spontaneous duplication and deletion rate in the *C. elegans* genome, nor in the MA populations within this study. Although this region may experience a higher than average duplication rate, this alone does not appear to account for the high frequency of individuals possessing this duplication within these independent populations. Mutation pressure (in this case, the spontaneous rate of CNV origin) is a very weak force in changing the frequency of alleles (or CNVs) [60]. The spontaneous duplication and deletion rates in *C. elegans* are on the order of 10^−7^/gene/generation [8]. Even after allowing for a 1000-fold higher rate of origin of a particular duplication than the best estimate of the spontaneous gene duplication rate, only 1 of 10,000 worms would incur that particular duplication in each generation and the expected frequency of a CNV containing a particular gene would reach 2 % by mutational input alone after 200 generations. Moreover, the spontaneous rate of duplication loss can be higher than the rate of origin of duplications and if we take the duplication loss rate into account, the rate of increase of a particular duplication in a population would be even slower and reach equilibrium rather than going to fixation or near fixation. Therefore, the rate of origin of CNVs alone cannot explain the observed increase in frequencies of CNVs in these populations.

## Conclusions

Our results demonstrate that gene copy-number changes can be a common class of adaptive genetic change to novel challenges in multicellular eukaryotes. Although the nature of the benefit that the CNVs provide in our experiments is still unknown, we note that these changes can arise frequently and sweep rapidly through populations. Some of these copy-number changes may be compensatory, serving to ameliorate the negative fitness consequences of deleterious mutations accrued during the mutation accumulation phase of the experiment. However, we note that many of these copy-number changes in our experimental populations may represent adaptations to the experimental laboratory conditions for the following reasons: (i) the presence of copy-number changes in control populations subjected to population expansion (adaptive recovery phase) without having undergone a previous fitness decline during mutation accumulation, (ii) convergent copy-number changes shared among adaptive recovery and control populations, and (iii) convergent copy-number changes in adaptive recovery populations descended from independent mutation accumulation lines. These results demonstrate the great potential that gene copy-number changes have for both adaptation *per se* as well as the potential for adaptive duplications as raw material for novel genes.

## Methods

### Base strain

The MA lines in this study were created with an obligately outcrossing, loss-of-function *fog-2* mutant strain of *C. elegans*. This strain was maintained as a frozen stock prior to the experiment. The *fog-2* locus in *C. elegans* is required for the initiation of spermatogenesis in hermaphrodites [61]. XX individuals homozygous for *fog-2* are transformed from self-fertile hermaphrodites to females whereas XO *fog-2* mutant males are indistinguishable from wild-type males. Therefore, a homozygous *fog-2* strain is fully competent as an outcrosser but not as a self-fertilizing hermaphroditic strain. The choice of outcrossing, rather than selfing, hermaphroditic populations to test if fitness recovery lines have high rates of duplications, was based on avoiding the effects of genetic hitch-hiking to the greatest extent possible [48].

### Creation of mutation accumulation lines by repeated bottlenecks and targeted RNAi knockdown of the mismatch repair gene msh-2

The MA phase of the experiment was initiated with a single male–female pair derived from the *fog-2(lf)* mutant line, kindly provided by the *Caenorhabditis* Genetics Center (St. Paul, MN). Four generations of single pair sib-matings were allowed from the resultant offspring to remove any freezer effects. From the F_5_ descendants of the base individual pair, 74 *fog-2(lf)*MA lines were initiated using a single female and two male siblings (Fig. 1a). The lines were assigned identification numbers 1 through 74, respectively. The presence of two males increased the probability of mating. The remaining siblings were expanded into thousands of worms and stored frozen at −80 °C for future use as a pre-MA ancestral control [62]. This pre-MA ancestral control served as a reference population to demonstrate potential fitness decline after MA.

The rate of spontaneous deleterious mutations in *C. elegans* is relatively low [63, 64], and it can take multiple years to see a significant fitness decline in the MA lines. In lieu of a spontaneous MA experiment, MA was independently accelerated in the experimental lines by simultaneously (i) bottlenecking populations, and (ii) reducing the functionality of the mismatch repair (MMR henceforth) gene *msh-2* by RNAi knockdown [65]. Silencing of the *msh-2* gene elevates mutation rates in the germline and somatic tissue of both sexes [66, 67]. A bacterial strain containing the feeding vector with the *msh-2* gene was obtained from Julie Ahringer at the University of Cambridge.

Each experimental line was subjected to 50 generations of MA, with bottlenecking and RNAi treatment at each generation. To ensure that mutations accumulated in the MA phase of the experiment were fixed within each line and not capable of segregation as wild-type alleles, each MA line was subjected to fifteen additional generations of full-sib mating without RNAi treatment. Treating the last MA generation as the reference population, fifteen generations of full-sib mating yields an inbreeding coefficient of 0.961 (*i.e.* 96.1 % reduction in heterozygosity relative to a random-mating subpopulation with the same allele frequencies) [68]. Thereafter, all extant MA lines were frozen at −80 °C.

### Population expansion of lines following mutation accumulation

After the MA phase, five MA lines with the greatest decline in fitness (MA7, 16, 19, 50, and 66) were each expanded into five populations (labeled A-E) and independently maintained at large population sizes under standard laboratory conditions [69]. To enable populations to expand to large sizes, the worms were housed on large 100 × 15 mm Petri dishes. Large population sizes were maintained across generations by transferring agar chunks to fresh plates with a sterilized scalpel every four days (equivalent to approximately one generation). This time period was adequate to ensure highly competitive conditions, as population sizes had reached several thousands of individuals prior to each transfer, with the animals being starved to the extent that egg-laying had ceased. To avoid cross-contamination between independent populations, petri plates were spaced apart on fiberglass trays and wrapped in parafilm. Populations were continually maintained at large population sizes for 180–212 generations (Fig. 1b). These large-population treatment adaptive recovery (RC) populations were frozen at −80 °C following ~80, ~130, ~ 180, and ~212 generations of large population treatment. For comparison, five control populations (C1 – C5) of *fog-2* were maintained at large population sizes for 208 generations without any prior MA treatment.

### Fitness assays during mutation accumulation and population expansion

During the MA phase, one fitness assay was conducted after 24 MA generations and the second after the termination of the MA phase (50 MA generations and 15 subsequent generations of full-sib mating without RNAi treatment). The fitness assay largely followed previous protocols for hermaphroditic MA lines [63] with minor modifications suited to outcrossing lines. The assays were conducted simultaneously on all extant MA lines, 25 adaptive recovery (RC) populations and five control populations (C1-C5) that had not been subjected to MA, but had been maintained at large populations sizes for the same period as the RC populations. The ancestral *fog-2* pre-MA ancestral population maintained as a frozen stock prior to the initiation of the MA experiment served as the control. The frozen ancestral control was thawed and 20 control lines were established independently from the surviving worms.

For fitness assays during the MA phase, a single sib-pair from each extant line was randomly chosen to enter the fitness assay. At the start of each assay, the 20 control and extant MA lines were expanded into five replicates (five individual sib-pair progeny of the ancestral pair), yielding 470 lines across both treatments. These 470 lines were maintained by transferring a sib-pair for two generations in the absence of RNAi to remove maternal effects. Additionally, because gene inactivation by RNAi does not appear to extend beyond the F_1_ generation [70], any decline in fitness in the MA lines should reflect mutation load due to heritable, germline mutations accumulated under the *msh-2* RNAi regime. Nonheritable, somatic mutations should not contribute to fitness decline once *msh-2* function is restored by RNAi termination, as these should not be inherited by the assayed individuals.

Productivity (the number of offspring produced) was measured using third generation individuals of the replicated control and experimental (MA, RC or C) populations. For each line, twelve L1 (first larval stage) F_3_ progeny were randomly selected upon hatching. After 36 h, surviving individuals had reached the L3-L4 larval stage at which they could be sexed. One male–female pair was randomly selected and transferred to a new petri dish for measuring productivity. Every 24 h ± 30 min thereafter, the focal sib-pair is transferred to a fresh plate. Daily transfers were terminated under the following conditions: (i) the female had not produced any eggs by day 8, or (ii) female mortality. Plates with eggs were placed at 20 °C for an additional 24 h period to enable hatching, then stored at 4 °C to kill the larvae for progeny counts. In order to score the number of offspring, the plates with dead progeny were stained with 0.0175 % Toluidine Blue to enable visualization of worms against the media. Productivity was calculated as the total number of progeny produced. The procedure was the same for the assay of adaptive RC and control (C1-C5) populations except that a random male–female pair was selected from each recovery population and control population to enter the fitness assay.

### Detection of CNVs via oligonucleotide array Comparative Genome Hybridization (oaCGH)

We analyzed copy-number changes in five MA lines (MA7, MA16, MA19, MA50 and MA66), 25 adaptive recovery populations (7A-E, 16A-E, 19A-E, 50A-E, 66A-E), and five additional control populations (C1-C5) that were propagated for the same period as the adaptive recovery populations but had not undergone a prior MA phase. In the microarray experiments, the MA lines and the C1–C5 populations were compared to their *fog-2* ancestor, and the adaptive recovery populations were compared to their post-MA ancestor (50 generations of MA and 15 generations of inbreeding). For example, copy number changes in recovery populations 7A–E were compared to MA7 after termination of the MA phase of the experiment. oaCGH analysis was performed as previously described [71]. We used oaCGH arrays manufactured by Roche NimbleGen Inc.: design 071114_CE2_WG_CGH_T, and new custom designed microarrays named 120618_Cele_WS230_JK_CGH. The new arrays are 3-plex microarrays with each individual sub-array comprising 720 k 50-mer oligonucleotide probes synthesized at random positions on the arrays. The filters used to select the probes primarily followed Maydan et al. [71] without focusing on coding regions in order to provide a more uniform coverage of the genome (Wormbase release WS230). In regions where unique probes could not be designed, selection filters were slightly relaxed in order to allow the inclusion of probes with possible cross-hybridization to at most one other location in the genome. The extraction of fluorescence intensity ratios and subsequent segmentation analysis followed Maydan et al. [71] closely except that a quantile normalization was applied on the log_2_ ratios. The segmentation algorithm used a bottom-up approach, adjacent segments being merged until no neighboring segments reach a user-defined similarity threshold, the similarity being calculated with a *t*-test. At the end of the segmentation procedure each remaining segment was analyzed and labeled as amplified/deleted if the log_2_ ratio values within the segment passed two user-defined filters, one for the average and one for the *p*-value (calculated with a *t*-test). Visual inspection of the log_2_ ratios was used to guide the selection of the three user-defined parameters applied to the automated segmentation procedure. Additional analyses were performed with JCFread_cgh (Matlab script), and SnoopCGH [72].

The minimum length of these CNVs was calculated based on the distance between the first and last probe inside the region that had been duplicated or deleted. The breakpoint of the CNVs is expected to be located between the first or last internal probe and the adjacent flanking probe. However, in some cases the distance between the adjacent flanking probes and the probes contained in the CNV was fairly large, up to 40 kb, resulting in uncertainty about the location of the breakpoints.

Additionally, we used (i) qPCR, (ii) PCR and DNA sequencing of breakpoints, and (iii) single-worm PCR to independently verify the presence of CNVs identified by oaCGH as well as quantify the frequency of the CNVs in earlier generations of the adaptive recovery phase.

### Quantitative PCR (qPCR)

We used qPCR as a means to independently verify the presence of CNVs identified by oaCGH as well as quantify the frequency of the CNVs in earlier generations of the adaptive recovery phase. The qPCR was performed and analyzed as described previously [8]. Briefly, qPCR was performed using FastStart SYBR Green with Rox (Roche) and the reactions were run on an ABI Prism 7000 Sequence Detection System. qPCR was done by testing population DNA of specified generations against their post-MA, pre-adaptive recovery ancestor.

A modification of the ΔΔCt method [73] was used for measurement of copy-number changes in genomic DNA from populations. The efficiency of the reference was determined by a dilution series for each qPCR plate. Each “run” was comprised of four groups of three unpaired technical replicates, one group for each combination of template and primers (reference DNA with reference primers (R/R’), reference DNA with test primers (R/T’), test DNA with reference primers (T/R’) and test DNA with test primers (T/T’)), resulting in 12 cycle threshold measurements (Cts) per run. The average of each group was used to calculate copy-number. The mean copy-number was determined from (1 + efficiency)^-ΔΔCt^ where ΔΔCt = (T/T’ – T/R’) – (R/T’ – R/R’) [74]. Statistical analysis was performed as recommended by MIQE standards [75]. 95 % confidence intervals for the mean copy-numbers were determined through bootstrapping (10,000 iterations) by random resampling of individual Ct values within each group to produce an array of sorted copy-numbers. The confidence interval bounds were the 2.5 and 97.5 % quantiles of the sorted bootstrap array.

### PCR and DNA sequencing across duplication and deletion breakpoints

For PCR and sequencing duplication breakpoints, we designed primers oriented in opposite directions within the predicted boundaries of the duplication event. In genomes bearing only a single gene-copy, the forward and reverse primers are divergent and would fail to initiate PCR amplification. However, in the event of gene duplication resulting in two adjacent paralogs (tandem or inverted), the primers are rendered convergent, enabling PCR amplification and subsequent DNA sequencing. For deletions, primers were designed to DNA sequences flanking the deleted sequence. This approach would fail to detect gene duplications and deletions with additional local rearrangements or those that have been rendered genomically distant via translocations. The PCR products were either gel-extracted and cleaned up using QIAquick Gel Extraction Kit (Qiagen) or prepared directly for sequencing using ExoSAP-IT (GE HealthCare Life Sciences). The PCR products were subsequently sequenced using Big Dye Terminator v3.1 Cycle Sequencing Kits (AB Applied Biosystems) on an ABI 3130xl Genetic Analyzer.

### Single-Worm PCR

Single-worm PCR was additionally performed to confirm the accuracy of both the oaCGH and qPCR methods in estimating the frequency of existing deletions and duplications. Because adaptive recovery populations were cryogenically frozen at multiple time-intervals approximating generations 80, 140, and 200, it was possible to resurrect *C. elegans* populations at different generation times and collect individual worms from the thawed populations. Populations at varying generation times were removed from −86 °C and thawed on regular NGM plates. Upon reaching maturity, worms were sexed and adult males were collected in lysis buffer and frozen in individual PCR tubes at −86 °C. It was necessary to use adult males because outcrossing adult females may contain nonclonal eggs; hence a PCR band of DNA extracted from a mother and her eggs would not be an accurate representation of the genotype of an individual worm. Using primers designed to detect duplications and deletions, PCR was performed on 30 individual worms, when possible, using the single-worm PCR protocol developed by Williams et al. [76]. Frozen males were thawed and incubated at 65 °C for 90′, followed by incubation at 95 °C for 15′ to deactivate proteinase K. After worms were lysed and DNA released from cells, PCR tubes were spun down to separate worm protein from solution. The DNA solution was removed from the tubes and divided between two PCR tubes, 2.5 *μl* per tube.

We obtained single-worm PCR data at varying generation times for rearrangements for which duplication/deletion breakpoints had previously been sequenced. On average, 30 individuals for each population at each time-point were analyzed. To test the frequency of a deletion in a population, two separate reactions were prepared, (i) namely using deletion primers external to the deleted sequence, and (ii) primers internal to the deleted sequence. A positive result for the reaction containing the internal primers was evidence that the deletion was not present in the genome of the individual. A positive result for the reaction with primers external to the deleted sequence was evidence that the deletion had occurred in the genome of the individual. The presence of both deletion single worm PCR products indicated an individual that was heterozygous for the deletion of interest. To estimate the frequency of duplication in a population, two reactions were prepared for each individual. One reaction was prepared with divergent primers designed from sequencing the breakpoints of the duplication in question and yields a product of a known size when the duplication is present, and the second reaction contained positive control primers. All reactions were run with a touchdown thermocycling protocol with the following profile: 10 cycles of 30s @ 94 °C, 30s @ 60 °C – 1 °C/cycle, and 2′ @ 72 °C followed by 30 cycles of 30s @ 94 °C, 30s @ 50 °C, and 2′ @ 72 °C. The products were analyzed by gel electrophoresis.

If the rearrangement resides on chromosome X, then the frequency of individuals showing a positive PCR result for the rearrangement should be a direct estimate of the frequency in the population since males are hemizygous for the X chromosome. If the rearrangement was present on any of the remaining five autosomes (I–V), the frequency of rearrangements was calculated under the assumption that the population was in Hardy-Weinberg equilibrium. The frequency of individuals that test negative for the rearrangement is therefore expected to be the frequency of individuals homozygous for the absence of the rearrangement (non-carriers). The frequency of individuals positive for the rearrangement is the frequency of individuals that are homozygous or heterozygous for the rearrangement. The frequency of the rearrangement is then estimated as 1 – square root of the frequency of non-carriers.

## Availability of data and materials

The microarray data have been deposited in NCBI’s Gene Expression Omnibus [77] and are accessible through GEO Series accession number GSE67871.

## Additional files

Additional file 1: Supplemental Data S1. List of ORFs contained in 25 duplications detected by oaCGH in five control and 25 adaptive recovery experimental *C. elegans* lines following 180–212 generations of population expansion under competitive conditions. The duplications are listed in Table 1. Duplication breakpoint coordinates and ORFs contained therein are based on Wormbase version WS243. (PDF 148 kb) Additional file 2: Supplemental Data S2. List of ORFs contained in 25 deletions detected by oaCGH in five control and 25 adaptive recovery experimental *C. elegans* lines following 180–212 generations of population expansion under competitive conditions. The deletions are listed in Table 2. Deletion breakpoint coordinates and ORFs contained therein are based on Wormbase version WS243. (PDF 104 kb) Additional file 3: Figure S1. Increase in the frequency of parallel duplication events in two populations containing an overlapping region on Chromosome II. The average copy-number per haploid genome was calculated from qPCR results and is indicated on the vertical axis. The number of recovery generations is indicated on the horizontal axis. (PDF 70 kb) Additional file 4: Figure S2. Increase in the frequency of parallel duplication events in two populations containing an overlapping region on Chromosome IV. The average copy-number per haploid genome was calculated from qPCR results and is indicated on the vertical axis. The number of recovery generations is indicated on the horizontal axis. (PDF 71 kb) Additional file 5: Figure S3. Increase in the frequencies of five unique duplications that lack overlap in their duplication spans. Frequencies of five unique duplications in adaptive recovery populations 7B, 16C, 50A, and 50D. The average copy-number per haploid genome was calculated from qPCR results and is indicated on the vertical axis. The number of recovery generations is indicated on the horizontal axis. (PDF 81 kb) Additional file 6: Figure S4. Increase in the frequencies of four unique duplications that lack overlap in their duplication spans. Frequencies of four unique duplications in adaptive recovery populations 19C, and 19E. The average copy-number per haploid genome was calculated from qPCR results and is indicated on the vertical axis. The number of recovery generations is indicated on the horizontal axis. (PDF 77 kb) Additional file 7: Figure S5. Increase in the frequencies of parallel deletion events in two control populations, C2 and C4, containing an overlapping region on Chromosome I. The average copy-number per haploid genome was calculated from qPCR results and is indicated on the vertical axis. The number of recovery generations is indicated on the horizontal axis. The results show a strong decline in average copy-number of these two independent deletions that were initially detected by oaCGH. The deletions have reached fixation when the average copy-number has reached 0. (PDF 70 kb) Additional file 8: Figure S6. Increase in the frequencies of parallel deletion events in three adaptive recovery populations (16A, 19A, and 19E), containing an overlapping region on Chromosome X. The average copy-number per haploid genome was calculated from qPCR results and is indicated on the vertical axis. The number of recovery generations is indicated on the horizontal axis. The results show a strong decline in average copy-number of these three independent deletions that were initially detected by oaCGH. The deletions have reached fixation when the average copy-number has reached 0. (PDF 78 kb) Additional file 9: Figure S7. Increase in the frequencies of parallel deletion events in two adaptive recovery populations (66D, and 66E) and one control population (C3) containing another overlapping region on Chromosome X. The average copy-number per haploid genome was calculated from qPCR results and is indicated on the vertical axis. The number of recovery generations is indicated on the horizontal axis. The results show a strong decline in average copy-number of these three independent deletions that were initially detected by oaCGH. The deletions have reached fixation when the average copy-number has reached 0. (PDF 76 kb) Additional file 10: Figure S8. Copy-number decreases for five unique deletion events in two adaptive recovery populations (66B, and 66D) that lack overlap in their deletion spans. The average copy-number per haploid genome was calculated from qPCR results and is indicated on the vertical axis. The generation from which the copy-number was estimated is indicated on the horizontal axis. The deletions have reached fixation when the average copy-number has reached 0. (PDF 82 kb) Additional file 11: Supplemental Data S3. List of ORFs contained in eight overlapping duplications and deletions in experimental *C. elegans* lines following 180–212 generations of population expansion under competitive conditions. Duplication/deletion breakpoint coordinates and ORFs contained therein are based on Wormbase version WS243. (PDF 116 kb)
