# Supplementary material for: Rapid Increase in frequency of gene copy-number variants during experimental evolution in Caenorhabditis elegans
Source: BMC Genomics. 2015 Dec 9;16:1044. doi: 10.1186/s12864-015-2253-2 (PMC4673709; doi:10.1186/s12864-015-2253-2)
Supplement: Additional file 1: Supplemental Data S1. — List of ORFs contained in 25 duplications detected by oaCGH in five control and 25 adaptive recovery experimental C. elegans lines following 180–212 generations of population expansion under competitive conditions. The duplications are listed in Table 1. Duplication breakpoint coordinates and ORFs contained therein are based on Wormbase version WS243. (PDF 148 kb) [file 12864_2015_2253_MOESM1_ESM.pdf]

Additional File 1: Supplemental Data S1

List of ORFs contained in 25 duplications detected by oaCGH in five control and 25 adaptive recovery experimental *C. elegans* lines following 180-212 generations of population expansion under competitive conditions. The duplications are listed in Table 1. Duplication breakpoint coordinates and ORFs contained therein are based on Wormbase version WS243.

Duplication in 7B:
Chr IV:6,837,045..6,879,487
Size = 42,443 bp
5 protein-coding genes:
lip-1 (C05B10.1), R13H7.2, *srx-20* (R13H7.1), *srx-19* (T05A12.1), *tre-2* (T05A12.2; partial duplication)
1 pseudogene:
R13H7.3

Duplication in 7B:
Chr V:19,505,848..20,101,145
Size = 595,298 bp
94 protein-coding genes:
Y43F8B.3 (partial duplication), Y43F8B.19, phy-4 (Y43F8B.4), Y43F8B.3, Y43F8B.2, Y43F8B.1, B0399.2, B0399.1, *nlp-25* (Y43F8C.1), Y43F8C.20, *oac-1* (B0399.2), *kcn1-1* (B0399.1), *nlp-25* (Y43F8C.1), *grsp-1* (Y43F8C.20), *nlp-26* (Y43F8C.2),Y43F8C.3, dyf-19 (Y43F8C.4), Y43F8C.5, Y43F8C.6, Y43F8C.7, *mrps-28* (Y43F8C.8), Y43F8C.9, *dmd-3* (Y43F8C.10), Y43F8C.11, *mrp-7* (Y43F8C.12), Y43F8C.13, *ani-3* (Y43F8C.14), Y43F8C.18, *srv-3* (Y43F8C.19), Y43F8C.15, Y43F8C.23, Y43F8C.16, Y43F8C.17, Y116F11A.6, Y116F11A.3, Y116F11A.1, W04E12.7, *fbxa-131* (W04E12.1), W04E12.2, W04E12.3, W04E12.4, W04E12.5, *clec-49* (W04E12.6), *clec-50* (W04E12.8), W04E12.9, M162.5, M162.15 *fbxa-118* (M162.8), *fbxa-194* (M162.11), *srt-45* (M162.3), *clec-258* (M162.2), M162.7, Y116F11B.2, *daf-28* (Y116F11B.1), Y116F11B.17, *pcp-4* (Y116F11B.3), *srw-38* (Y116F11B.5), Y116F11B.6, Y116F11B.7, Y116F11B.8, Y116F11B.1, *gly-4* (Y116F11B.12), Y116F11B.13, *fars-2* (Y60A3A.13), Y116F11B.14, *chk-2* (Y60A3A.12), Y60A3A.19, Y60A3A.16, *skr-4* (Y60A3A.18), Y60A3A.14, *dhs-24* (Y60A3A.10), Y60A3A.9, Y60A3A.8, Y60A3A.7, *srh-172* (Y60A3A.6), *srh-171* (Y60A3A.5), *srh-173* (Y60A3A.4), *srh-183* (Y60A3A.3), Y60A3A.24, *clec-260* (Y60A3A.2), *sri-67* (Y60A3A.22), Y60A3A.25, *unc-51* (Y60A3A.1), Y60A3A.23, Y60A3A.21, *lgc-55* (Y113G7A.5), Y113G7A.16, *spe-19* (Y113G7A.10), *srh-233* (Y113G7A.1), *ttx-1* (Y113G7A.6), *fre-1* (Y113G7A.8), *dcs-1* (Y113G7A.9), *sec-23* (Y113G7A.3), Y113G7A.15 (partial duplication)
43 pseudogenes:
B0399.t16, B0399.t15, B0399.t14, B0399.t1, B0399.t13, B0399.t12, B0399.t2, B0399.t4, B0399.t3, B0399.t5, B0399.t11, B0399.t10, B0399.t9, B0399.t8, B0399.t7, B0399.t6, Y43F8C.t1, Y43F8C.t9, Y43F8C.t2, Y43F8C.t8, Y43F8C.t3, Y43F8C.t4, Y43F8C.t7, Y43F8C.t6, Y43F8C.t5, Y43F8C.24, Y116F11A.4, W04E12.10,

M162.12, M162.13, M162.9, M162.4, M162.14, M162.6, Y116F11B.4, Y116F11B.10, Y60A3A.17, Y60A3A.15, Y60A3A.10, Y60A3A.28, Y60A3A.t1, Y60A3A.t2, Y113G7A.2

Duplication in 7D:
Chr IV:505,050..701,113
Size = 196,064 bp
38 protein-coding genes + 3 tRNA genes:
W03G1.5, *pig-1* (W03G1.6), *asm-3* (W03G1.7), W03G1.2, W03G1.8, *glt-7* (W03G1.1), F09C11.1, F56A11.4, *efn-4* (F56A11.3), F56A11.7, F56A11.5, *gex-2* (F56A11.1), F56A11.6, C18H7.12, C18H7.5, C18H7.6, C18H7.4, C18H7.7, C18H7.11, *srt-59* (C18H7.8), *prmt-4* (C18H7.9), *col-102* (C18H7.3), *inx-18* (C18H7.2), C18H7.1, *nhr-76* (C05G6.2), K11H12.9, K11H12.1, *rpl-15* (K11H12.2), K11H12.8, K11H12.7, K11H12.6, K11H12.11, K11H12.3, K11H12.4, K11H12.10, K11H12.5, *cut1-28* (F41A4.1), *cut1-26* (Y55F3C.7) (partial duplication)
1 pseudogene:
Y55F3C.17

Duplication in 16B*:
Chr V: 19,295,123..19,839,705
Size = 544,583 bp
110 protein-coding genes:
F55C9.6 (partial duplication), *fbxb-60* (F55C9.7), F55C9.14, *fbxb-62* (F55C9.8), *fbxb-63* (F55C9.13), *fbxb-61* (F55C9.10), F55C9.15, F55C9.11, C43D7.8, *fbxb-64* (C43D7.9), *srh-208* (C43D7.6), C43D7.7, *sdz-6* (C43D7.5), C43D7.4, *fbxb-65* (C43D7.2), C14B4.2, Y43F8A.1, Y43F8A.2, Y43F8A.3, *srw-84* (Y43F8A.4), Y43F8A.5, C25F9.8, C25F9.13, *srw-86* (C25F9.7), C25F9.12, C25F9.6, C25F9.10, C25F9.5, C25F9.4, C25F9.9, C25F9.15, C25F9.2, *srw-85* (C25F9.1), C25F9.11, C25F9.16, C25F9.14, M04C3.1, M04C3.2, M04C3.5, Y43F8B.14, Y43F8B.13, Y43F8B.24, Y43F8B.15, Y43F8B.25, Y43F8B.23, Y43F8B.12, Y43F8B.11, Y43F8B.10, Y43F8B.9, Y43F8B.22, Y43F8B.17,Y43F8B.28, Y43F8B.18, Y43F8B.7, Y43F8B.29, *scl-21* (Y43F8B.5), Y43F8B.3, Y43F8B.19, *phy-4* (Y43F8B.4), Y43F8B.2, Y43F8B.1, Y43F8B.20, *oac-1* (B0399.2), *kcnl-1* (B0399.1), *nlp-25* (Y43F8C.1), *grsp-1* (Y43F8C.20), *nlp-26* (Y43F8C.2), Y43F8C.3, *dyf-19* (Y43F8C.4), Y43F8C.5, Y43F8C.6, Y43F8C.7, mrps-28 (Y43F8C.8), Y43F8C.9, *dmd-3* (Y43F8C.10), Y43F8C.11, *mrp-7* (Y43F8C.12), Y43F8C.13, *ani-3* (Y43F8C.14), Y43F8C.18, *srv-3* (Y43F8C.19), Y43F8C.15, Y43F8C.23, Y43F8C.16, Y43F8C.17, Y116F11A.6, Y116F11A.3, Y116F11A.1, W04E12.7, *fbxa-131* (W04E12.1), W04E12.2, W04E12.3, W04E12.4, W04E12.5, *clec-49* (W04E12.6), *clec-50* (W04E12.8), W04E12.9, M162.5, M162.15, *fbxa-118* (M162.8), *fbxa-194* (M162.11), *srt-45* (M162.3), *clec-258* (M162.2), *clec-259* (M162.1), M162.7, Y116F11B.2, *daf-28* (Y116F11B.1), Y116F11B.17, *pcp-4* (Y116F11B.3), *srw-38* (Y116F11B.5)
52 pseudogenes:
C43D7.10, C43D7.11, C43D7.12, C43D7.3, C43D7.1, C14B4.t1, Y43F8A.t1, C25F9.t3, C25F9.t2, C25F9.t1, C25F9.t4, C25F9.t5, Y43F8B.8, Y43F8B.21,

Y43F8B.6, B0399.t16, B0399.t15, B0399.t14, B0399.t1, B0399.t13, B0399.t12, B0399.t2, B0399.t3, B0399.t4, B0399.t5, B0399.t11, B0399.t10, B0399.t9, B0399.t8, B0399.t7, B0399.t6, Y43F8C.t1, Y43F8C.t9, Y43F8C.t2, Y43F8C.t8, Y43F8C.26, Y43F8C.t3, Y43F8C.t4, Y43F8C.t7, Y43F8C.t6, Y43F8C.t5, Y43F8C.24, Y116F11A.4, W04E12.10, M162.12, M162.13, M162.9, M162.4, M162.14, M162.6, Y116F11B.4, *srz-35* (Y116F11B.4) (partial duplication)

Duplication in 16C:
Chr IV:9,054,304..9,457,751
Size = 403,448 bp
89 protein-coding genes:

nhr-11 (ZC410.1), *mppb-1* (ZC410.2), *mans-4* (ZC410.3), *twk-8* (ZC410.4), ZC410.5, *1pl-1* (ZC410.7), *icln-1* (C01F6.8), C01F6.9, *cpna-3* (C01F6.1), C01F6.2, C01F6.14, *fem-3* (C01F6.4), *aly-1* (C01F6.5), *nrfl-1* (C01F6.6), *delm-1* (F23B2.3), *daf-10* (F23B2.4), *flp-1* (F23B2.5), *rpb-12* (F23B2.13), *aly-2* (F23B2.6), F23B2.7, F23B2.10, *pcp-3* (F23B2.11), *pcp-2* (F23B2.12), C07C7.3, C07C7.1, C46C2.5, C46C2.7, *wnk-1* (C46C2.1), C46C2.6, C46C2.2, C46C2.3, Y11D7A.3, *rab-28* (Y11D7A.4), Y11D7A.5, Y11D7A.7, Y11D7A.8, Y11D7A.9, Y11D7A.10, *col-120* (Y11D7A.11), *flh-1* (Y11D7A.12), Y11D7A.19, *flh-13* (Y11D7A.13), *hum-9* (Y11D7A.14), *nhr-267* (H22D14.1), *nhr-264* (F14A5.1), F49C12.1, F49C12.2, F49C12.3, F49C12.4, F49C12.5, F49C12.6, F49C12.7, F49C12.9, *rpn-7* (F49C12.8), F49C12.10, F49C12.11, F49C12.12, *vha-17* (F49C12.13), F49C12.14, F49C12.15, *CLEC-183* (T20D3.1), T20D3.2, T20D3.3, T20D3.5, T20D3.6, *vps-26* (T20D3.7), T20D3.8, T20D3.11, C10C5.1, C10C5.2, C10C5.3, C10C5.4, C10C5.5, C10C5.7, *daf-15* (C10C5.6), *col-121* (F56D5.1), F56D5.2, F56D5.3, F56D5.6, F56D5.5, F56D5.9, *srxa-2* (F56D5.10), F59B8.1, *idh-1* (F59B8.2), F38E11.9, *hsp-12.3* (F38E11.1), *hsp-12.6* (F38E11.2), *cut1-17* (F38E11.4), *cpin-1* (F38E11.3)

8 pseudogenes:

F23B2.9, F23B2.8, C46C2.4, Y11D7A.1, Y11D7A.16, *srg-52* (Y11D7A.18), F56D5.4, F56D5.8

Duplication in 16C:
Chr V:800,408..1,103,333
Size = 302,926 bp
57 protein-coding genes:

nhr-270 (R13D11.8), R13D11.11, R13D11.4, R13D11.10, R13D11.3, R13D11.1, *srx-32* (R13D11.9), *srx-31* (F41H8.4), F41H8.2, F41H8.1, K09C6.7, K09C6.10, K09C6.8, K09C6.6, *srbc-13* (K09C6.5), *srbc-12* (K09C6.4), K09C6.3, K09C6.9, K09C6.2, K09C6.1, T02B11.3, T02B11.4, T02B11.9, T02B11.8, *srg-53* (T02B11.1), *srj-38* (T02B11.5), T02B11.6, T02B11.10, *nas-32* (T02B11.7), *fmo-5* (H24K24.5), H24K24.4, H24K24.3, H24K24.2, Y50D4C.2, Y50D4C.3, Y50D4C.6, *sqv-6* (Y50D4C.4), *unc-34* (Y50D4C.1), Y50D4C.5, *ergo-1* (R09A1.1), R09A1.2, R09A1.3, *flp-34* (R09A1.5), *nra-4* (C02E11.1), K10C9.4, K10C9.9, *str-224* (K10C9.8), K10C9.3, *str-67* (K10C9.6), K10C9.7, K10C9.1, Y50D4B.7, Y50D4B.6, *clec-203* (Y50D4B.5), Y50D4B.4, Y50D4B.3, Y50D4B.2 (partial duplication)

2 pseudogenes:

srx-30 (F41H8.3), *str-53* (T02B11.2),

Duplication in 16D:
Chr II: 6,248,049..6,406,772
Size = 158,724 bp
<u>48 protein-coding genes:</u>
T24H7.3 (partial duplication), T24H7.2, *phb-2* (T24H7.1), F13H8.5, F13H8.11, *nmgp-1* (F13H8.4), F13H8.12, F13H8.3, F13H8.8, F13H8.2, *bpl-1* (F13H8.10), F13H8.9, F13H8.1, F13H8.6, F13H8.7, C29F5.3, *mps-1* (C29F5.4), C29F5.5, *sdz-3* (C29F5.2), C29F5.1, C29F5.8, *glb-10* (C29F5.7), C32D5.3, C32D5.4, *sma-6* (C32D5.2), *set-4* (C32D5.5), C32D5.6, C32D5.14, C32D5.7, C32D5.8, C32D5.1, *lgg-1* (C32D5.9), C32D5.10, C32D5.11, C32D5.12, K10B2.4, *ani-2* (K10B2.5), *clec-88* (K10B2.3), K10B2.2, *lin-23* (K10B2.1), F58F12.1, F58F12.4, F58F12.2, F58F12.3, *zig-10* (T25D10.2), *btb-2* (T25D10.5), T25D10.1, *spp-11* (T25D10.3) (partial duplication)
<u>1 pseudogene:</u>
K10B2.t1

Duplication in 16D:
Chr V: 19,746,828..19,885,746
Size = 138,919 bp
<u>26 protein-coding genes:</u>
W04E12.4 (partial duplication), W04E12.5, *clec-49* (W04E12.6), *clec-50* (W04E12.8), W04E12.9, M162.5, M162.15, *fbxa-118* (M162.8), *fbxa-194* (M162.11), *srt-45* (M162.3), *clec-258* (M162.2), *clec-259* (M162.1), M162.7, Y116F11B.2, *daf-28* (Y116F11B.1), Y116F11B.17, *pcp-4* (Y116F11B.3), *srw-38* (Y116F11B.5), Y116F11B.6, Y116F11B.7, Y116F11B.9, Y116F11B.8, Y116F11B.9a, Y116F11B.11, *gly-4* (Y116F11B.12), Y116F11B.13 (partial duplication)
<u>8 pseudogenes:</u>
M162.12, M162.13, M162.9, *srt-46* (M162.4), M162.14, M162.6, *srz-35* (Y116F11B.4), Y116F11B.10

Duplication in 16E*:
Chr V:19,295,580..19,840,162
Size = 544,583 bp
<u>110 protein-coding genes:</u>
F55C9.6 (partial duplication), *fbxb-60* (F55C9.7), F55C9.14, *fbxb-62* (F55C9.8), *fbxb-63* (F55C9.13), *fbxb-61* (F55C9.10), F55C9.15, F55C9.11, C43D7.8, *fbxb-64* (C43D7.9), *srh-208* (C43D7.6), C43D7.7, *sdz-6* (C43D7.5), C43D7.4, *fbxb-65* (C43D7.2), C14B4.2, Y43F8A.1, Y43F8A.2, Y43F8A.3, *srw-84* (Y43F8A.4), Y43F8A.5, C25F9.8, C25F9.13, *srw-86* (C25F9.7), C25F9.12, C25F9.6, C25F9.10, C25F9.5, C25F9.4, C25F9.9, C25F9.15, C25F9.2, *srw-85* (C25F9.1), C25F9.11, C25F9.16, C25F9.14, M04C3.1, M04C3.2, M04C3.5, Y43F8B.14, Y43F8B.13, Y43F8B.24, Y43F8B.15, Y43F8B.25, Y43F8B.23, Y43F8B.12, Y43F8B.11, Y43F8B.10, Y43F8B.9, Y43F8B.22, Y43F8B.17,Y43F8B.28, Y43F8B.18, Y43F8B.7, Y43F8B.29, *scl-21* (Y43F8B.5), Y43F8B.3, Y43F8B.19, *phy-4*

(Y43F8B.4), Y43F8B.2, Y43F8B.1, Y43F8B.20, *oac-1* (B0399.2),kcnl-1 (B0399.1), *nlp-25* (Y43F8C.1), *grsp-1* (Y43F8C.20), *nlp-26* (Y43F8C.2), Y43F8C.3, *dyf-19* (Y43F8C.4), Y43F8C.5, Y43F8C.6, Y43F8C.7, *mrps-28* (Y43F8C.8), Y43F8C.9, *dmd-3* (Y43F8C.10), Y43F8C.11, *mrp-7* (Y43F8C.12), Y43F8C.13, *ani-3* (Y43F8C.14), Y43F8C.18, *srv-3* (Y43F8C.19), Y43F8C.15, Y43F8C.23, Y43F8C.16, Y43F8C.17, Y116F11A.6, Y116F11A.3, Y116F11A.1, W04E12.7, *fbxa-131* (W04E12.1), W04E12.2, W04E12.3, W04E12.4, W04E12.5, *clec-49* (W04E12.6), *clec-50* (W04E12.8), W04E12.9, M162.5, M162.15, *fbxa-118* (M162.8), *fbxa-194* (M162.11), *srt-45* (M162.3), *clec-258* (M162.2), *clec-259* (M162.1), M162.7, Y116F11B.2, *daf-28* (Y116F11B.1), Y116F11B.17, *pcp-4* (Y116F11B.3), *srw-38* (Y116F11B.5)

<u>52 pseudogenes:</u>

C43D7.10, C43D7.11, C43D7.12, C43D7.3, C43D7.1, C14B4.t1, Y43F8A.t1, C25F9.t3, C25F9.t2, C25F9.t1, C25F9.t4, C25F9.t5, Y43F8B.8, Y43F8B.21, Y43F8B.6, B0399.t16, B0399.t15, B0399.t14, B0399.t1, B0399.t13, B0399.t12, B0399.t2, B0399.t3, B0399.t4, B0399.t5, B0399.t11, B0399.t10, B0399.t9, B0399.t8, B0399.t7, B0399.t6, Y43F8C.t1, Y43F8C.t9, Y43F8C.t2, Y43F8C.t8, Y43F8C.26, Y43F8C.t3, Y43F8C.t4, Y43F8C.t7, Y43F8C.t6, Y43F8C.t5, Y43F8C.24, Y116F11A.4, W04E12.10, M162.12, M162.13, M162.9, M162.4, M162.14, M162.6, Y116F11B.4, *srz-35* (Y116F11B.4) (partial duplication)

Duplication in 19C:

Chr V:7,637,941..7,641,911

Size = 3,971 bp

<u>3 protein-coding genes:</u>

clec-46 (F07C4.9) (partial duplication), *clec-45* (F07C4.2), F07C4.10

<u>0 pseudogenes:</u>

Duplication in 19C:

Chr II:14,037,517.. 14,039,164

Size = 7,572 bp

<u>1 protein-coding genes:</u>

daf-45 (W01G7.1) (partial duplication)

<u>0 pseudogenes:</u>

Duplication in 19E:

Chr X:813,802.. 821,373

Size = 7,572 bp

<u>2 protein-coding genes:</u>

ifd-2 (F25E2.4), *daf-3* (F25E2.5) (partial duplication)

<u>0 pseudogenes:</u>

Duplication in 19E:

Chr X:829,580.. 835,392

Size = 5,813 bp

<u>2 protein-coding genes:</u>

F39H12.2, F39H12.1 (partial duplication)
0 pseudogenes:

Duplication in 50A:
Chr V:19,780,484.. 19,972,052
Size = 191,569 bp
30 protein-coding genes:
srt-45 (M162.3), *clec-258* (M162.2), *clec-259* (M162.1), M162.7, Y116F11B.2, *daf-28* (Y116F11B.1), Y116F11B.17, *pcp-4* (Y116F11B.3), *srw-38* (Y116F11B.5), Y116F11B.6, Y116F11B.7, Y116F11B.9, Y116F11B.8, Y116F11B.9a, Y116F11B.11, *gly-4* (Y116F11B.12), Y116F11B.13, *fars-2* (Y60A3A.13), Y116F11B.14, *chk-2* (Y60A3A.12), Y60A3A.19, Y60A3A.16, *skr-4* (Y60A3A.18), Y60A3A.14, *dhs-24* (Y60A3A.10), Y60A3A.9, Y60A3A.8, Y60A3A.7, *srh-172* (Y60A3A.6), *srh-171* (Y60A3A.5) (partial duplication)
8 pseudogenes:
srt-46 (M162.4), M162.14, M162.6, *srz-35* (Y116F11B.4), Y116F11B.10, Y60A3A.17, *nhr-240* (Y60A3A.15), Y60A3A.11

Duplication in 50A:
Chr X:8,624,771..9,024,484
Size = 399,714 bp
64 protein-coding genes:
K01A12.3 (partial duplication), *stg-2* (F12D9.1), F12D9.2, *rig-1* (K09E2.4), K09E2.2, K09E2.3, K09E2.1, *frpr-8* (K09E2.5), *jbts-14* (F53A9.4), F53A9.3, F53A9.2, F53A9.1, F53A9.6, F53A9.7, F53A9.8, F53A9.9, *tnt-2* (F53A9.10), EGAP4.1, M02D8.6, M02D8.3, M02D8.2, M02D8.7, *asns-2* (M02D8.4), M02D8.5, M02D8.1, ZK271.4, ZK271.3, *unc-27* (ZK271.2), *chup-1* (ZK271.1), R04E5.7, R04E5.8, R04E5.9, R04E5.2, *ifd-1* (R04E5.10), C28G1.5. C28G1.6, *sec-15* (C28G1.3), C28G1.2, *ubc-23* (C28G1.1), C28G1.10, C28G1.4, C06E2.5, C06E2.9, *ins-9* (C06E2.8), *ubc-22* (C06E2.7), *ubc-21* (C06E2.3), C06E2.1, C06E2.2, C13E3.1, D1009.3, *cyn-8* (D1009.2), *nlp-14* (D1009.4), *acs-2* (D1009.1), *dylt-2* (D1009.5), D1073.1, *aexr-3* (C48C5.3), *nmur-1* (C48C5.1), *twk-18* (C24A3.6), C24A3.4, C24A3.2, C24A3.1, C24A3.9, T25B6.4, T25B6.5
0 pseudogenes:

Duplication in 50B:
Chr V:19,781,064.. 19,972,507
Size = 191,444 bp
30 protein-coding genes:
srt-45 (M162.3), *clec-258* (M162.2), *clec-259* (M162.1), M162.7, Y116F11B.2, *daf-28* (Y116F11B.1), Y116F11B.17, *pcp-4* (Y116F11B.3), *srw-38* (Y116F11B.5), Y116F11B.6, Y116F11B.7, Y116F11B.9, Y116F11B.8, Y116F11B.9a, Y116F11B.11, *gly-4* (Y116F11B.12), Y116F11B.13, *fars-2* (Y60A3A.13), Y116F11B.14, *chk-2* (Y60A3A.12), Y60A3A.19, Y60A3A.16, *skr-4* (Y60A3A.18), Y60A3A.14, *dhs-24* (Y60A3A.10), Y60A3A.9, Y60A3A.8, Y60A3A.7, *srh-172* (Y60A3A.6), *srh-171* (Y60A3A.5)

8 pseudogenes:

srt-46 (M162.4), M162.14, M162.6, *srz-35* (Y116F11B.4), Y116F11B.10, Y60A3A.17, *nhr-240* (Y60A3A.15), Y60A3A.11

Duplication in 50C:

Chr V:19,659,829.. 19,976,506

Size = 316,680 bp

58 protein-coding genes:

Y43F8C.11, *mrp-7* (Y43F8C.11), Y43F8C.13, *ani-3* (Y43F8C.14), Y43F8C.18, *srv-3* (Y43F8C.19), Y43F8C.15, Y43F8C.23, Y43F8C.16, Y43F8C.17, Y116F11A.6, Y116F11A.3, Y116F11A.1, W04E12.7, *fbxa-131* (W04E12.1), W04E12.2, W04E12.3, W04E12.4, W04E12.5a, W04E12.5b, *clec-49* (W04E12.6), *clec-50* (W04E12.8), W04E12.9, M162.5, M162.15, *fbxa-118* (M162.8), *fbxa-194* (M162.11), *srt-45* (M162.3), *clec-258* (M162.2), *clec-259* (M162.1), M162.7, Y116F11B.2, *daf-28* (Y116F11B.1), Y116F11B.17, *pcp-4* (Y116F11B.13), *srw-38* (Y116F11B.5), Y116F11B.6, Y116F11B.7, Y116F11B.8, Y116F11B.9a, Y116F11B.11, *gly-4* (Y116F11B.12), Y116F11B.13, *fars-2* (Y60A3A.13), Y116F11B.14, *cchk-2* (Y60A3A.12), Y60A3A.19, Y60A3A.16, *skr-4* (Y60A3A.18), Y60A3A.14, *dhs-24* (Y60A3A.10), Y60A3A.9, Y60A3A.8, Y60A3A.7, *srh-172* (Y60A3A.6), *srh-171* (Y60A3A.5), *srh-173* (Y60A3A.4), *srh-183* (Y60A3A.3) (partial duplication)

20 pseudogenes:

Y43F8C.t7, Y43F8C.t6, Y43F8C.t5, Y43F8C.24, Y116F11A.4, W04E12.10, M162.12, M162.13, M162.9, *srt-46* (M162.4), M162.14, M162.6, *srz-35* (Y116F11B.4), Y116F11B.10, Y60A3A.17, *nhr-240* (Y60A3A.15), Y60A3A.17, *nhr-240* (Y60A3A.15), Y60A3A.11, Y60A3A.28

Duplication in 50D:

Chr IV:560,240.. 1,024,886

Size = 464,647 bp

84 protein-coding genes:

efn-4 (F56A11.3), F56A11.7, F56A11.5, *gex-2* (F56A11.1), F56A11.6, C18H7.12, C18H7.5, C18H7.6, C18H7.4, C18H7.7, C18H7.11, *srt-59* (C18H7.8), *prmt-4* (C18H7.9), *col-102* (C18H7.3), *inx-18* (C18H7.2), C18H7.1, *nhr-76* (C05G6.2), K11H12.9, K11H12.1, *rpl-15* (K11H12.2), K11H12.8, K11H12.7, K11H12.6, K11H12.11, K11H12.3, K11H12.4, K11H12.10, K11H12.5, *cut1-28* (F41A4.1), *cut1-26* (Y55F3C.7), *clec-164* (Y55F3C.5), Y55F3C.10, Y55F3C.9, *srt-24* (Y55F3C.8), *kvs-5* (Y55F3C.3), *srt-23* (Y55F3C.2), *gst-40* (F56B3.10), *col-103* (F56B3.1), F56B3.2, F56B3.3, F56B3.9, *mrpl-2* (F56B3.8), *ugt-52* (F56B3.7), F56B3.4, F56B3.6, *skr-18* (F56B3.12), F56B3.11, *ech-5* (F56B3.5), *mrpl-46* (Y55F3BL.1), Y55F3BL.4, Y55F3BL.6, Y55F3BL.2, *madf-1* (Y55F3BR.5), Y55F3BR.10, Y55F3BR.6, Y55F3BR.7, *lgc-33* (Y55F3BR.4), *lem-4* (Y55F3BR.8), Y55F3BR.11, Y55F3BR.2, Y55F3BR.1, *mak-2* (C44C8.6), *fbxc-1* (C44C8.4), *fbxc-9* (C44C8.10), *fbxc-2* (C44C8.3), *fbxc-10* (C44C8.9), *fbxc-4* (C44C8.2), *fbxc-11* (C44C8.8), *fbxc-5* (C44C8.1), *fbxc-12* (C44C8.7), *fbxc-3* (F58H7.8), *fbxc-8* (F58H7.7), F58H7.5, *lgc-30* (F58H7.3), F58H7.1, *faah-3* (F58H7.2), *plx-1*

(Y55F3AL.1), *egrh-2* (Y55F3AM.7), Y55F3AM.6, Y55F3AM.5, *immp-2* (Y55F3AM.8), Y55F3AM.9, *atg-3* (Y55F3AM.4), Y55F3AM.3

2 pseudogenes:

Y55F3C.17, Y55F3C.13,

Duplication in 50D:

Chr V:18,703,541..18,723,878

Size = 20,338 bp

4 protein-coding genes:

Y69H2.9 (partial duplication), Y17D7C.1, Y17D7C.6, Y17D7C.2

5 pseudogenes:

Y69H2.18, Y69H2.16, Y17D7C.5, Y17D7C.4, Y17D7C.3

Duplication in 50D:

Chr V:19,780,935..19,966,260

Size = 185,326 bp

30 protein-coding genes:

srt-45 (M162.3), *clec-258* (M162.2), *clec-259* (M162.1), M162.7, Y116F11B.2, *daf-28* (Y116F11B.1), Y116F11B.17, *pcp-4* (Y116F11B.3), *srw-38* (Y116F11B.5), Y116F11B.6, Y116F11B.7, Y116F11B.9, Y116F11B.8, Y116F11B.9a, Y116F11B.11, *gly-4* (Y116F11B.12), Y116F11B.13, *fars-2* (Y60A3A.13), Y116F11B.14, *chk-2* (Y60A3A.12), Y60A3A.19, Y60A3A.16, *skr-4* (Y60A3A.18), Y60A3A.14, *dhs-24* (Y60A3A.10), Y60A3A.9, Y60A3A.8, Y60A3A.7, *srh-172* (Y60A3A.6), *srh-171* (Y60A3A.5) (partial duplication)

8 pseudogenes:

srt-46 (M162.4), M162.14, M162.6, *srz-35* (Y116F11B.4), Y116F11B.10, Y60A3A.17, Y60A3A.15, Y60A3A.11

Duplication in 50E:

Chr II:6,312,598..6,444,674

Size = 132,077 bp

32 protein-coding genes:

C32D5.3, C32D5.4, *sma-6* (C32D5.2), *set-4* (C32D5.5), C32D5.6, C32D5.14, C32D5.7, C32D5.8, C32D5.1, *lgg-1* (C32D5.9), C32D5.10, C32D5.11, C32D5.12, K10B2.4, *ani-2* (K10B2.5), *clec-88* (K10B2.3), K10B2.2, *lin-23* (K10B2.1), F58F12.1, F58F12.4, F58F12.2, F58F12.3, *zig-10* (T25D10.2), *btb-2* (T25D10.5), T25D10.1, *spp-11* (T25D10.3), T25D10.4, K03H9.3, *col-75* (K03H9.2), K03H9.1, *cutl-16* (K06A1.3), K06A1.2 (partial duplication)

1 pseudogene:

K10B2.t1

Duplication in 50E:

Chr V:19,780,952..19,966,162

Size = 185,211 bp

30 protein-coding genes:

srt-45 (M162.3), *clec-258* (M162.2), *clec-259* (M162.1), M162.7, Y116F11B.2, *daf-28* (Y116F11B.1), Y116F11B.17, *pcp-4* (Y116F11B.3), *srw-38* (Y116F11B.5), Y116F11B.6, Y116F11B.7, Y116F11B.9, Y116F11B.8, Y116F11B.9a, Y116F11B.11, *gly-4* (Y116F11B.12), Y116F11B.13, *fars-2* (Y60A3A.13), Y116F11B.14, *chk-2* (Y60A3A.12), Y60A3A.19, Y60A3A.16, *skr-4* (Y60A3A.18), Y60A3A.14, *dhs-24* (Y60A3A.10), Y60A3A.9, Y60A3A.8, Y60A3A.7, *srh-172* (Y60A3A.6), *srh-171* (Y60A3A.5) (partial duplication)

8 pseudogenes:

srt-46 (M162.4), M162.14, M162.6, *srz-35* (Y116F11B.4), Y116F11B.10, Y60A3A.17, *nhr-240* (Y60A3A.15), Y60A3A.11

Duplication in 66C:

Chr V:19,393,526..20,054,330

Size = 660,805 bp

121 protein-coding genes:

C25F9.8, C25F9.13, *srw-86* (C25F9.7), C25F9.12, C25F9.6, C25F9.10, C25F9.5, C25F9.4, C25F9.9, C25F9.15, C25F9.2, *srw-85* (C25F9.1), C25F9.11, C25F9.16, C25F9.14, M04C3.1, Y43F8B.14, Y43F8B.13, Y43F8B.24, Y43F8B.15, Y43F8B.25, Y43F8B.23, Y43F8B.12, Y43F8B.11, Y43F8B.10, Y43F8B.9, Y43F8B.22, Y43F8B.17, Y43F8B.28, Y43F8B.18, Y43F8B.7, Y43F8B.29, *sc1-21* (Y43F8B.5), Y43F8B.3, Y43F8B.19, *phy-4* (Y43F8B.4b), Y43F8B.2, Y43F8B.1, Y43F8C.20, *oac-1* (B0399.2), *kcnl-1* (B0399.1), *nlp-25* (Y43F8C.1), *grsp-1* (Y43F8C.2 0), *nlp-26* (Y43F8C.2), Y43F8C.3, *dyf-19* (Y43F8C.4), Y43F8C.5, Y43F8C.6, Y43F8C.7, *mrps-28* (Y43F8C.8), Y43F8C.9, *dmd-3* (Y43F8C.10), Y43F8C.11, *mrp-7* (Y43F8C.12), Y43F8C.13, *ani-3* (Y43F8C.14), Y43F8C.18, *srv-3* (Y43F8C.19), Y43F8C.15, Y43F8C.23, Y43F8C.16, Y43F8C.17, Y116F11A.6, Y116F11A.3, Y116F11A.1, W04E12.7, *fbxa-131* (W04E12.1), W04E12.2, W04E12.3, W04E12.4, W04E12.5, *clec-49* (W04E12.6), *clec-50* (W04E12.8), W04E12.9, M162.5, M162.15, *fbxa-118* (M162.8), *fbxa-194* (M162.11), *srt-45* (M162.3), *clec-258* (M162.2), *clec-259* (M162.1), M162.7, Y116F11B.2, *daf-28* (Y116F11B.1), Y116F11B.17, *pcp-4* (Y116F11B.3), *srw-38* (Y116F11B.5), Y116F11B.6, Y116F11B.7, Y116F11B.8, Y116F11B.9a, Y116F11B.11, *gly-4* (Y116F11B.12), Y116F11B.13, *fars-2* (Y60A3A.13), Y116F11B.14, *chk-2* (Y60A3A.12), Y60A3A.19, Y60A3A.16, *skr-4* (Y60A3A.18), Y60A3A.14, *dhs-24* (Y60A3A.10), Y60A3A.9, Y60A3A.8, Y60A3A.7, *srh-172* (Y60A3A.6), *srh-171* (Y60A3A.5), *srh-173* (Y60A3A.4), *srh-183* (Y60A3A.3), Y60A3A.24, *clec-260* (Y60A3A.2), *sri-67* (Y60A3A.22), Y60A3A.25, *unc-51* (Y60A3A.1), Y60A3A.23, Y60A3A.21, *lgc-55* (Y113G7A.5), Y113G7A.16, *spe-19* (Y113G7A.10), *srh-233* (Y113G7A.1), *ttx-1* (Y113G7A.6) (partial duplication)

52 pseudogenes:

Y43F8A.t1, C25F9.t3, C25F9.t2, C25F9.t1, C25F9.t4, C25F9.t5, Y43F8B.8, Y43F8B.21, Y43F8B.6, B0399.t16, B0399.t15, B0399.t14, B0399.t1, B0399.t13, B0399.t12, B0399.t2, B0399.t4, B0399.t3, B0399.t5, B0399.t11, B0399.t10, B0399.t9, B0399.t8, B0399.t7, B0399.t6, Y43F8C.t1, Y43F8C.t9, Y43F8C.t2, Y43F8C.t8, Y43F8C.t3, Y43F8C.t4, Y43F8C.t7, Y43F8C.t6, Y43F8C.t5, Y43F8C.24, Y116F11A.4, W04E12.10, M162.12, M162.13, M162.9, M162.4,

M162.14, M162.6, Y116F11B.4, Y116F11B.10, Y60A3A.17, Y60A3A.15, Y60A3A.11, Y60A3A.28, Y60A3A.t1, Y60A3A.t2, Y113G7A.2

Duplication in 66E:
Chr V:19,295,300..19,839,882
Size = 544,583 bp
<u>111 protein-coding genes:</u>
F55C9.6 (partial duplication), *fbxb-60* (F55C9.7), F55C9.14, *fbxb-62* (F55C9.8), *fbxb-63* (F55C9.13), *fbxb-61* (F55C9.10), F55C9.11, F55C9.15, C43D7.8, *fbxb-64* (C43D7.9), *srh-208* (C43D7.6), C43D7.7, *sdz-6* (C43D7.5), C43D7.4, *fbxb-65* (C43D7.2), C14B4.2, Y43F8A.1, Y43F8A.2, Y43F8A.3, *srw-84* (Y43F8A.4), Y43F8A.5, C25F9.8, C25F9.13, *srw-86* (C25F9.7), C25F9.12, C25F9.6, C25F9.10, C25F9.5, C25F9.4, C25F9.9, C25F9.15, C25F9.2, *srw-85* (C25F9.1), C25F9.11, C25F9.16, C25F9.14, M04C3.1, M04C3.2, M04C3.5, Y43F8B.14, Y43F8B.13, Y43F8B.24, Y43F8B.15, Y43F8B.25, Y43F8B.23, Y43F8B.12, Y43F8B.11, Y43F8B.10, Y43F8B.9, Y43F8B.22, Y43F8B.17, Y43F8B.28, Y43F8B.18, Y43F8B.7, Y43F8B.29, *scl-21* (Y43F8B.5), Y43F8B.3, Y43F8B.19, *phy-4* (Y43F8B.4), Y43F8B.2, Y43F8B.1, Y43F8B.20, *oac-1* (B0399.2), *kcnl-1* (B0399.1), *nlp-25* (Y43F8C.1), *grsp-1* (Y43F8C.20), *nlp-26* (Y43F8C.2), Y43F8C.3, *dyf-19* (Y43F8C.4), Y43F8C.5, Y43F8C.6, Y43F8C.7, *mrps-28* (Y43F8C.8), Y43F8C.9, *dmd-3* (Y43F8C.10), Y43F8C.11, *mrp-7* (Y43F8C.12), Y43F8C.13, *ani-3* (Y43F8C.14), Y43F8C.18, *srv-3* (Y43F8C.19), Y43F8C.15, Y43F8C.23, Y43F8C.16, Y43F8C.17, Y116F11A.6, Y116F11A.3, Y116F11A.1, W04E12.7, *fbxa-131* (W04E12.1), W04E12.2, W04E12.3, W04E12.4, W04E12.5, *clec-49* (W04E12.6), *clec-50* (W04E12.8), W04E12.9, M162.5, M162.15, *fbxa-118* (M162.8), *fbxa-194* (M162.11), *srt-5* (M162.3), *clec-258* (M162.2), *clec-259* (M162.1), M162.7, Y116F11B.2, *daf-28* (Y116F11B.1), Y116F11B.17, *pcp-4* (Y116F11B.3), *srw-38* (Y116F11B.5)
<u>50 pseudogenes:</u>
C43D7.10, C43D7.11, C43D7.12, C43D7.3, C43D7.1, C14B4.t1, Y43F8A.t1, C25F9.t3, C25F9.t2, C25F9.t1, C25F9.t4, C25F9.t5, Y43F8B.8, Y43F8B.21, Y43F8B.6, B0399.t16, B0399.t15, B0399.t14, B0399.t1, B0399.t13, B0399.t12, B0399.t2, B0399.t4, B0399.t3, B0399.t5, B0399.t11, B0399.t10, B0399.t9, B0399.t8, B0399.t7, B0399.t6, Y43F8C.t1, Y43F8C.t9, Y43F8C.t2, Y43F8C.t8, Y43F8C.t3, Y43F8C.t4, Y43F8C.t7, Y43F8C.t6, Y43F8C.t5, Y43F8C.24, Y116F11A.4, W04E12.10, M162.12, M162.13, M162.9, M162.4, M162.14, M162.6, Y116F11B.4 (partial duplication)

Duplication in C2:
Chr V:19,295,101..19,839,683
Size = 544,583 bp
<u>111 protein-coding genes:</u>
F55C9.6 (partial duplication), *fbxb-60* (F55C9.7), F55C9.14, *fbxb-62* (F55C9.8), *fbxb-63* (F55C9.13), *fbxb-61* (F55C9.10), F55C9.11, F55C9.15, C43D7.8, *fbxb-64* (C43D7.9), *srh-208* (C43D7.6), C43D7.7, *sdz-6* (C43D7.5), C43D7.4, *fbxb-65* (C43D7.2), C14B4.2, Y43F8A.1, Y43F8A.2, Y43F8A.3, *srw-84* (Y43F8A.4),

Y43F8A.5, C25F9.8, C25F9.13, *srw-86* (C25F9.7), C25F9.12, C25F9.6, C25F9.10, C25F9.5, C25F9.4, C25F9.9, C25F9.15, C25F9.2, *srw-85* (C25F9.1), C25F9.11, C25F9.16, C25F9.14, M04C3.1, M04C3.2, M04C3.5, Y43F8B.14, Y43F8B.13, Y43F8B.24, Y43F8B.15, Y43F8B.25, Y43F8B.23, Y43F8B.12, Y43F8B.11, Y43F8B.10, Y43F8B.9, Y43F8B.22, Y43F8B.17, Y43F8B.28, Y43F8B.18, Y43F8B.7, Y43F8B.29, *scl-21* (Y43F8B.5), Y43F8B.3, Y43F8B.19, *phy-4* (Y43F8B.4), Y43F8B.2, Y43F8B.1, Y43F8B.20, *oac-1* (B0399.2), *kcnl-1* (B0399.1), *nlp-25* (Y43F8C.1), *grsp-1* (Y43F8C.20), *nlp-26* (Y43F8C.2), Y43F8C.3, *dyf-19* (Y43F8C.4), Y43F8C.5, Y43F8C.6, Y43F8C.7, *mrps-28* (Y43F8C.8), Y43F8C.9, *dmd-3* (Y43F8C.10), Y43F8C.11, *mrp-7* (Y43F8C.12), Y43F8C.13, *ani-3* (Y43F8C.14), Y43F8C.18, *srv-3* (Y43F8C.19), Y43F8C.15, Y43F8C.23, Y43F8C.16, Y43F8C.17, Y116F11A.6, Y116F11A.3, Y116F11A.1, W04E12.7, *fbxa-131* (W04E12.1), W04E12.2, W04E12.3, W04E12.4, W04E12.5, *clec-49* (W04E12.6), *clec-50* (W04E12.8), W04E12.9, M162.5, M162.15, *fbxa-118* (M162.8), *fbxa-194* (M162.11), *srt-5* (M162.3), *clec-258* (M162.2), *clec-259* (M162.1), M162.7, Y116F11B.2, *daf-28* (Y116F11B.1), Y116F11B.17, *pcp-4* (Y116F11B.3), *srw-38* (Y116F11B.5)

50 pseudogenes:

C43D7.10, C43D7.11, C43D7.12, C43D7.3, C43D7.1, C14B4.t1, Y43F8A.t1, C25F9.t3, C25F9.t2, C25F9.t1, C25F9.t4, C25F9.t5, Y43F8B.8, Y43F8B.21, Y43F8B.6, B0399.t16, B0399.t15, B0399.t14, B0399.t1, B0399.t13, B0399.t12, B0399.t2, B0399.t4, B0399.t3, B0399.t5, B0399.t11, B0399.t10, B0399.t9, B0399.t8, B0399.t7, B0399.t6, Y43F8C.t1, Y43F8C.t9, Y43F8C.t2, Y43F8C.t8, Y43F8C.t3, Y43F8C.t4, Y43F8C.t7, Y43F8C.t6, Y43F8C.t5, Y43F8C.24, Y116F11A.4, W04E12.10, M162.12, M162.13, M162.9, M162.4, M162.14, M162.6, Y116F11B.4 (partial duplication)
