# Supplementary material for: Rapid Increase in frequency of gene copy-number variants during experimental evolution in Caenorhabditis elegans
Source: BMC Genomics. 2015 Dec 9;16:1044. doi: 10.1186/s12864-015-2253-2 (PMC4673709; doi:10.1186/s12864-015-2253-2)
Supplement: Additional file 2: Supplemental Data S2. — List of ORFs contained in 25 deletions detected by oaCGH in five control and 25 adaptive recovery experimental C. elegans lines following 180–212 generations of population expansion under competitive conditions. The deletions are listed in Table 2. Deletion breakpoint coordinates and ORFs contained therein are based on Wormbase version WS243. (PDF 104 kb) [file 12864_2015_2253_MOESM2_ESM.pdf]

Additional File 2: Supplemental Data S2

List of ORFs contained in 25 deletions detected by oaCGH in five control and 25 adaptive recovery experimental *C. elegans* lines following 180-212 generations of population expansion under competitive conditions. The deletions are listed in Table 2. Deletion breakpoint coordinates and ORFs contained therein are based on Wormbase version WS243.

Deletion in 16A*:
Chr X:817,573..830,086
Size = 12,514 bp
1 protein-coding genes:
daf-3 (F25E2.5)

Deletion in 16D*:
Chr V:7,663,133..7,687,447
Size = 24,315 bp
7 protein-coding genes:
C12D5.5, C12D5.4, C12D5.3, *cyp-33A1* (C12D5.7), *sre-11* (C12D5.11), *nhr-94* (C12D5.8), *nhr-152* (C12D5.2) (partial deletion)

Deletion in 19A*:
Chr X:800,773..827,100
Size = 26,328 bp
5 protein-coding genes:
gtr-1 (F25E2.1), F25E2.2, F25E2.3, *ifd-2* (F25E2.4), *daf-3* (F25E2.5)

Deletion in 19C:
Chr V:7,642,395..7,682,740
Size = 40,346 bp
10 protein-coding genes:
F07C4.11, *str-47* (F07C4.1), F07C4.12, *srh-234* (F07C4.14), *srh-200* (F07C4.13), C12D5.5, C12D5.4, C12D5.3, *cyp-33A1* (C12D5.7), *sre-11* (C12D5.11)

Deletion in 19E:
Chr X:821,499..829,454
Size = 7,956 bp
1 protein-coding genes:
daf-3 (F25E2.5) (partial deletion)

Deletion in 50B:
Chr V:7,650,284..7,693,435
Size = 43,152 bp
12 protein-coding genes:

F07C4.12 (partial deletion), *srh-234* (F07C4.14), *srh-200* (F07C4.13), C12D5.5, C12D5.4, C12D5.3, *cyp-33A1* (C12D5.7), *sre-11* (C12D5.11), *nhr-94* (C12D5.8), *nhr-152* (C12D5.2), C12D5.9, C12D5.10

<u>1 Pseudogene:</u>

str-147 (C12D5.1)

Deletion in 50C:

Chr V: 7,647,125..7,696,096

Size = 48,972 bp

<u>14 protein-coding genes:</u>

str-47 (F07C4.1), F07C4.12, *srh-234* (F07C4.14), *srh-200* (F07C4.13), C12D5.5, C12D5.4, C12D5.3, *cyp-33A1* (C12D5.7), *sre-11* (C12D5.11), *nhr-94* (C12D5.8), *nhr-152* (C12D5.2), C12D5.9, C12D5.10, ZK105.3 (partial deletion)

<u>1 Pseudogene:</u>

str-147 (C12D5.1)

Deletion in 50C:

Chr X: 1,029..273,082

Size = 272,054 bp

<u>35 protein-coding genes:</u>

CE7X_3.1, Y73B3A.1, Y73B3A.20, Y73B3A.18, Y73B3A.3, Y73B3A.4, *elk-2* (Y73B3A.5), *fbxa-221* (Y73B3A.15), *fbxa-222* (Y73B3A.22), *fbxa-16* (Y73B3A.14), Y73B3A.13, Y73B3A.7, *cal-6* (Y73B3A.12), Y73B3A.8, Y73B3A.11, Y73B3A.9, Y73B3A.10, T08D2.1, T08D2.4, T08D2.5, T08D2.6, T08D2.7, T08D2.8, Y73B3B.1, Y73B3B.3, *set-28* (Y73B3B.2), AC8.4, AC8.3, AC8.7, AC8.11, AC8.10, AC8.12, *set-33* (Y108F1.3), *math-43* (Y108F1.4), Y108F1.5 (partial deletion)

<u>18 Pseudogenes:</u>

cTel7X.1, CE7X_3.2, CE7X_3.4, Y35H6.3, Y73B3A.21, Y73B3A.2, Y73B3A.17, Y73B3A.16, Y73B3A.t1, T08D2.9, T08D2.2, T08D2.3, Y73B3B.5, AC8.6, AC8.5, AC8.9, pme-6 (AC8.1), AC8.2

Deletion in 50D*:

Chr V:7,653,667..7,680,465

Size = 26,799 bp

<u>6 protein-coding genes:</u>

srh-234 (F07C4.14) (partial deletion), *srh-200* (F07C4.13), C12D5.5, C12D5.4, C12D5.3, *cyp-33A1* (C12D5.7) (partial deletion)

Deletion in 50D:

Chr X:1,029..295,671

Size = 294,643 bp

<u>38 protein-coding genes:</u>

CE7X_3.1, Y73B3A.1, Y73B3A.20, Y73B3A.18, Y73B3A.16, Y73B3A.3, Y73B3A.4, *elk-2* (Y73B3A.5), *fbxa-221* (Y73B3A.15), *fbxa-222* (Y73B3A.22), *fbxa-16* (Y73B3A.14), Y73B3A.13, Y73B3A.7, *cal-6* (Y73B3A.12), Y73B3A.8, Y73B3A.11, Y73B3A.9, Y73B3A.10, T08D2.1, T08D2.4, T08D2.5, T08D2.6,

T08D2.7, T08D2.8, Y73B3B.1, Y73B3B.3, *set-28* (Y73B3B.2), AC8.4, AC8.3, AC8.7, AC8.11, AC8.10, AC8.12, *set-33* (Y108F1.3), *math-43* (Y108F1.4), Y108F1.5, Y108F1.1, Y47C4A.1

20 Pseudogenes:

cTel7X.1, CE7X_3.2, CE7X_3.4, Y35H6.3, Y73B3A.21, Y73B3A.2, Y73B3A.17, Y73B3A.16, Y73B3A.t1, T08D2.9, T08D2.2, T08D2.3, Y73B3B.5, AC8.6, AC8.5, AC8.9, *pme-6* (AC8.1), AC8.2, Y47C4A.t1, Y47C4A.t2

Deletion in 50E*:

Chr V:7,652,044..7,682,914

Size = 30,871 bp

8 protein-coding genes:

F07C4.12B (partial deletion), *srh-234* (F07C4.14), *srh-200* (F07C4.13), C12D5.5, C12D5.4, C12D5.3, *cyp-33A1* (C12D5.7), *sre-11* (C12D5.11) (partial deletion)

Deletion in 66B:

Chr V:15,258,727..15,326,180

Size = 67,454 bp

26 protein-coding genes:

srsx-37 (M01B2.7), M01B2.8, M01B2.10, M01B2.12, M01B2.13, T10H4.13, *srw-22* (T10H4.3), T10H4.4, *srw-16* (T10H4.5), *srw-17* (T10H4.6), *srw-19* (T10H4.8), *srx-51* (T10H4.9), *cyp-34A1* (T10H4.10), *cyp-34A2* (T10H4.11), *str-96* (T10H4.2), *cpr-3* (T10H4.12), *srx-48* (T26H8.2), T26H8.5, T26H8.4, *srz-10* (ZK1037.11), *irld-62* (ZK1037.1), *srt-22* (ZK1037.3), *nhr-246* (ZK1037.4), ZK1037.13, *nhr-247* (ZK1037.5), ZK1037.6 (partial deletion)

5 Pseudogenes:

srw-18 (T10H4.7), T10H4.1, *srx-49* (T26H8.3), ZK1037.12, ZK1037.2

Deletion in 66B*:

Chr X:9,983,441..9,999,107

Size = 15,667 bp

2 protein-coding genes:

F19C6.5, *grk-1* (F19C6.1) (partial deletion)

Deletion in 66D:

Chr V:18,665,661..18,670,354

Size = 4,694 bp

1 protein-coding gene:

Y69H2.10 (partial deletion)

Deletion in 66D:

Chr V:18,701,820..18,725,404

Size = 23,585 bp

3 protein-coding genes:

nhr-241 (Y69H2.8) (partial deletion), Y69H2.9, Y17D7C.1, Y17D7C.6, Y17D7C.2

5 Pseudogenes:

Y69H2.18, Y69H2.16, Y17D7C.5, Y17D7C.4, Y17D7C.3

Deletion in 66D:
Chr X:961,361..963,014
Size = 1,654 bp
1 protein-coding gene:
ncs-1 (C44C1.3) (partial deletion)

Deletion in 66D:
Chr X:7,528,608..7,529,729
Size = 1,122 bp
1 protein-coding gene:
ceh-14 (F46C8.5) (partial deletion)

Deletion in 66E:
Chr X:7,528,608..7,529,729
Size = 1,122 bp
1 protein-coding gene:
ceh-14 (F46C8.5) (partial deletion)

Deletion in C1:
Chr I:15,060,622..15,071,438
Size = 10,817 bp
0 protein-coding genes
4 rRNA genes:
F31C3.7, F31C3.11, F31C3.9, F31C3.8
1 Pseudogene:
rrn-3.56 (F31C3.10)

Deletion in C2:
Chr I:15,060,388..15,071,427
Size = 11,040 bp
0 protein-coding genes
4 rRNA genes:
F31C3.7, F31C3.11, F31C3.9, F31C3.8
1 Pseudogene:
rrn-3.56 (F31C3.10)

Deletion in C3:
Chr II:14,034,460..14,039,471
Size = 5,012 bp
1 protein-coding gene:
daf-5 (W01G7.1) (partial deletion)

Deletion in C3*:
Chr X:7,527,813..7,529,236

Size = 1,424 bp

1 protein-coding gene:

ceh-14 (F46C8.5) (partial deletion)

Deletion in C4:

Chr I:15,060,388..15,071,427

Size = 11,040 bp

0 protein-coding genes

4 rRNA genes:

F31C3.7, F31C3.11, F31C3.9, F31C3.8

1 Pseudogene:

rrn-3.56 (F31C3.10)

Deletion in C5:

Chr I:15,061,973..15,071,438

Size = 9,466 bp

0 protein-coding genes

4 rRNA genes:

F31C3.7, F31C3.11, F31C3.9, F31C3.8

Deletion in C5:

Chr X:823,167..827,286

Size = 4,120 bp

1 protein-coding gene:

daf-3 (F25E2.5) (partial deletion)
