# Supplementary material for: Rapid Increase in frequency of gene copy-number variants during experimental evolution in Caenorhabditis elegans
Source: BMC Genomics. 2015 Dec 9;16:1044. doi: 10.1186/s12864-015-2253-2 (PMC4673709; doi:10.1186/s12864-015-2253-2)
Supplement: Additional file 4: Figure S2. — Increase in the frequency of parallel duplication events in two populations containing an overlapping region on Chromosome IV. The average copy-number per haploid genome was calculated from qPCR results and is indicated on the vertical axis. The number of recovery generations is indicated on the horizontal axis. (PDF 71 kb) [file 12864_2015_2253_MOESM4_ESM.pdf]

Additional File 4: Suppl Figure S2

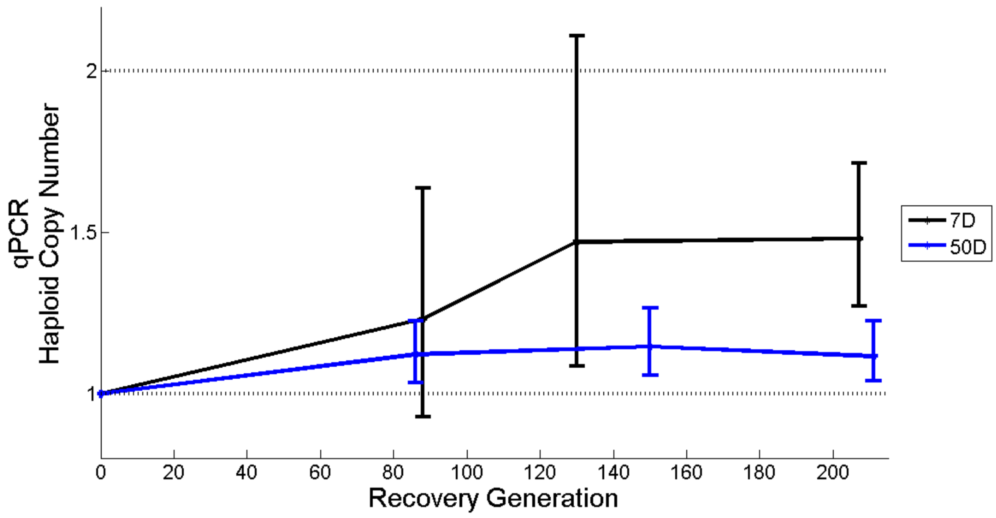

Supplemental Figure S2. Increase in the frequency of parallel duplication events in two populations containing an overlapping region on Chromosome IV. The average copy-number per haploid genome was calculated from qPCR results and is indicated on the vertical axis. The number of recovery generations is indicated on the horizontal axis.
