# Supplementary material for: Rapid Increase in frequency of gene copy-number variants during experimental evolution in Caenorhabditis elegans
Source: BMC Genomics. 2015 Dec 9;16:1044. doi: 10.1186/s12864-015-2253-2 (PMC4673709; doi:10.1186/s12864-015-2253-2)
Supplement: Additional file 5: Figure S3. — Increase in the frequencies of five unique duplications that lack overlap in their duplication spans. Frequencies of five unique duplications in adaptive recovery populations 7B, 16C, 50A, and 50D. The average copy-number per haploid genome was calculated from qPCR results and is indicated on the vertical axis. The number of recovery generations is indicated on the horizontal axis. (PDF 81 kb) [file 12864_2015_2253_MOESM5_ESM.pdf]

**Additional File 5: Suppl Figure S3**

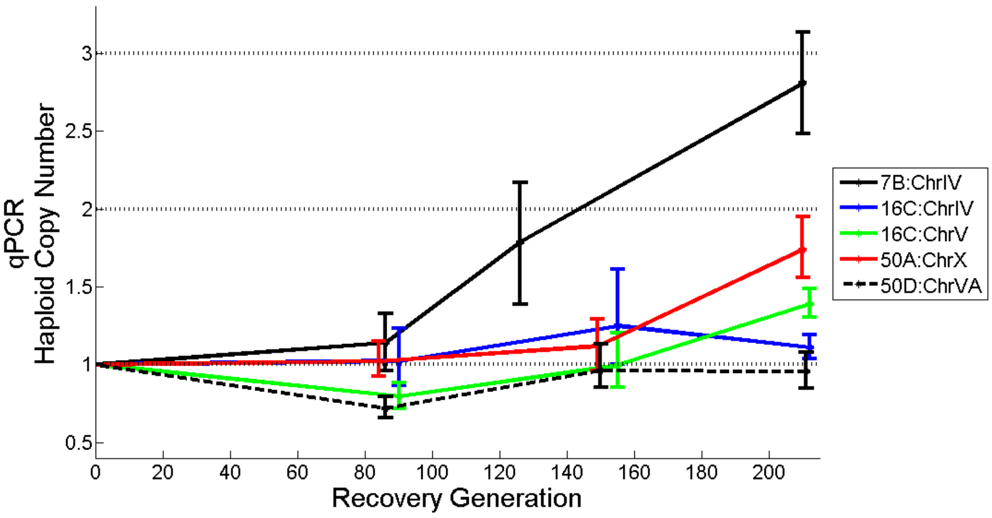


**Supplemental Figure S3.** Increase in the frequencies of five unique duplications that lack overlap in their duplication spans. Frequencies of five unique duplications in adaptive recovery populations 7B, 16C, 50A, and 50D. The average copy-number per haploid genome was calculated from qPCR results and is indicated on the vertical axis. The number of recovery generations is indicated on the horizontal axis.
