# Supplementary material for: Rapid Increase in frequency of gene copy-number variants during experimental evolution in Caenorhabditis elegans
Source: BMC Genomics. 2015 Dec 9;16:1044. doi: 10.1186/s12864-015-2253-2 (PMC4673709; doi:10.1186/s12864-015-2253-2)
Supplement: Additional file 6: Figure S4. — Increase in the frequencies of four unique duplications that lack overlap in their duplication spans. Frequencies of four unique duplications in adaptive recovery populations 19C, and 19E. The average copy-number per haploid genome was calculated from qPCR results and is indicated on the vertical axis. The number of recovery generations is indicated on the horizontal axis. (PDF 77 kb) [file 12864_2015_2253_MOESM6_ESM.pdf]

Additional File 6: Suppl Figure S4

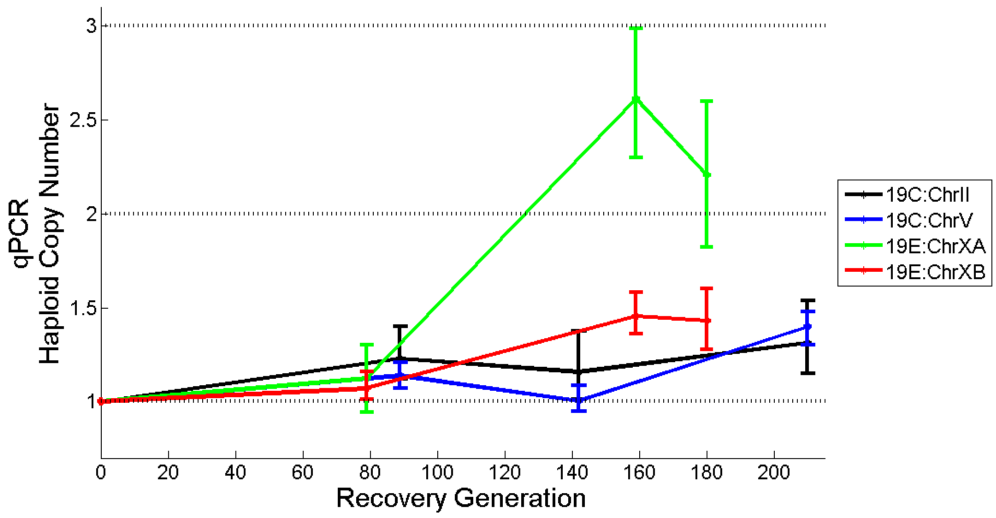

Supplemental Figure S4. Increase in the frequencies of four unique duplications that lack overlap in their duplication spans. Frequencies of four unique duplications in adaptive recovery populations 19C, and 19E. The average copy-number per haploid genome was calculated from qPCR results and is indicated on the vertical axis. The number of recovery generations is indicated on the horizontal axis.
