# Supplementary material for: Rapid Increase in frequency of gene copy-number variants during experimental evolution in Caenorhabditis elegans
Source: BMC Genomics. 2015 Dec 9;16:1044. doi: 10.1186/s12864-015-2253-2 (PMC4673709; doi:10.1186/s12864-015-2253-2)
Supplement: Additional file 8: Figure S6. — Increase in the frequencies of parallel deletion events in three adaptive recovery populations (16A, 19A, and 19E), containing an overlapping region on Chromosome X. The average copy-number per haploid genome was calculated from qPCR results and is indicated on the vertical axis. The number of recovery generations is indicated on the horizontal axis. The results show a strong decline in average copy-number of these three independent deletions that were initially detected by oaCGH. The deletions have reached fixation when the average copy-number has reached 0. (PDF 78 kb) [file 12864_2015_2253_MOESM8_ESM.pdf]

Additional File 8: Suppl Figure S6

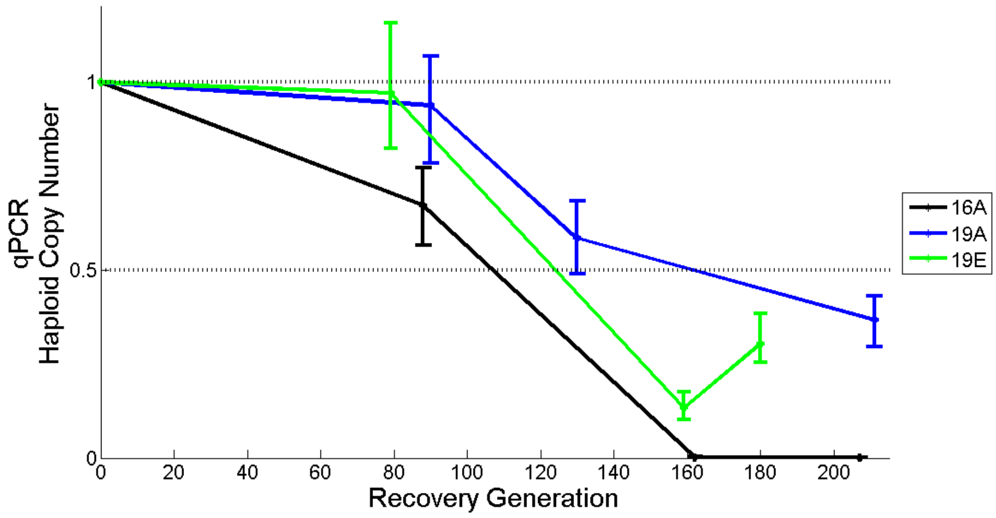

Supplemental Figure S6. Increase in the frequencies of parallel deletion events in three adaptive recovery populations (16A, 19A, and 19E), containing an overlapping region on Chromosome X. The average copy-number per haploid genome was calculated from qPCR results and is indicated on the vertical axis. The number of recovery generations is indicated on the horizontal axis. The results show a strong decline in average copy-number of these three independent deletions that were initially detected by oaCGH. The deletions have reached fixation when the average copy-number has reached 0.
