# Supplementary material for: Rapid Increase in frequency of gene copy-number variants during experimental evolution in Caenorhabditis elegans
Source: BMC Genomics. 2015 Dec 9;16:1044. doi: 10.1186/s12864-015-2253-2 (PMC4673709; doi:10.1186/s12864-015-2253-2)
Supplement: Additional file 10: Figure S8. — Copy-number decreases for five unique deletion events in two adaptive recovery populations (66B, and 66D) that lack overlap in their deletion spans. The average copy-number per haploid genome was calculated from qPCR results and is indicated on the vertical axis. The generation from which the copy-number was estimated is indicated on the horizontal axis. The deletions have reached fixation when the average copy-number has reached 0. (PDF 82 kb) [file 12864_2015_2253_MOESM10_ESM.pdf]

Additional File 10: Suppl Figure S8

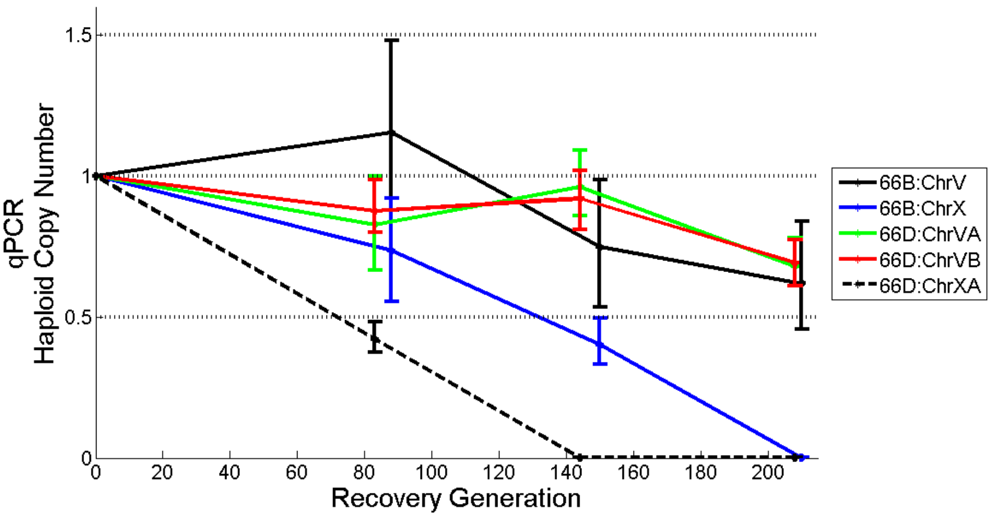

Supplemental Figure S8. Copy-number decreases for five unique deletion events in two adaptive recovery populations (66B, and 66D) that lack overlap in their deletion spans. The average copy-number per haploid genome was calculated from qPCR results and is indicated on the vertical axis. The generation from which the copy-number was estimated is indicated on the horizontal axis. The deletions have reached fixation when the average copy-number has reached 0.
