# Supplementary material for: Rapid Increase in frequency of gene copy-number variants during experimental evolution in Caenorhabditis elegans
Source: BMC Genomics. 2015 Dec 9;16:1044. doi: 10.1186/s12864-015-2253-2 (PMC4673709; doi:10.1186/s12864-015-2253-2)
Supplement: Additional file 11: Supplemental Data S3. — List of ORFs contained in eight overlapping duplications and deletions in experimental C. elegans lines following 180–212 generations of population expansion under competitive conditions. Duplication/deletion breakpoint coordinates and ORFs contained therein are based on Wormbase version WS243. (PDF 116 kb) [file 12864_2015_2253_MOESM11_ESM.pdf]

Additional File 11: Supplemental Data S3

List of ORFs contained in eight overlapping duplications and deletions in experimental *C. elegans* lines following 180-212 generations of population expansion under competitive conditions. Duplication/deletion breakpoint coordinates and ORFs contained therein are based on Wormbase version WS243.

Overlapping Duplications:

1. Chromosome II:
   - 16D: 6,248,049..6,406,772
   - 50E: 6,312,598..6,444,674

   Overlapping region: 6,312,598-6,406,772 = 94,175 bp

   26 protein-coding ORFs

   - C32D5.3
     - Biological process: apoptotic process; embryo development ending in birth or egg hatching; receptor mediated reproduction
   - C32D5.4
     - Unclassified
   - *sma-6* (C32D5.2)
     - Biological process: BMP signaling pathway; body morphogenesis; dauer larval development; defense response to fungus; innate immune response; maintenance of protein location in nucleus; positive regulation of multicellular organism growth; positive regulation of protein catabolic process; positive regulation of transcription from RNA polymerase II promoter; protein phosphorylation; regulation of cell adhesion; regulation of cell morphogenesis; reproduction; tail tip morphogenesis
     - Cellular component: membrane; plasma membrane
     - Molecular functions: ATP binding; BMP binding; protein kinase activity; transforming growth factor beta-activated receptor activity; transmembrane receptor protein serine/threonine kinase activity
   - *set-4* (C32D5.5)
     - Biological process: determination of adult lifespan; embryo development ending in birth or egg-hatching
     - Molecular functions: protein binding
   - C32D5.6
     - Biological process: cellular response to DNA damage stimulus
     - Molecular functions: protein binding
   - C32D5.14
     - Unclassified
   - C32D5.7
     - Unclassified
   - C32D5.8
     - Biological process: embryo development ending in birth or egg-

hatching

C32D5.1

Unclassified

lgg-1 (C32D5.9)

Biological process: autophagy; dauer larval development; determination of adult lifespan; embryo development; embryo development ending in birth or egg-hatching; growth; necrotic cell death; positive regulation of necrotic cell death; programmed cell death

Cellular component: autophagic vacuole; autophagic vacuole membrane; cytoplasm; nucleus

C32D5.10

Biological process: nematode larval development; reproduction

Molecular function: metal ion binding; protein binding; zinc ion binding

C32D5.11

Biological process: apoptotic process; lipid storage

Molecular function: protein binding; zinc ion binding

C32D5.12

Biological process: body morphogenesis; embryo development ending in birth or egg-hatching; locomotion; nematode larval development; oxidation-reduction process; steroid biosynthetic process

Molecular function: 3-beta-hydroxy-delta5-steroid dehydrogenase activity; oxidoreductase activity, acting on the CH-OH group of donors, NAD or NADP as receptor

K10B2.4

Unclassified

ani-2 (K10B2.5)

Biological process: gonad development; embryo development ending in birth or egg hatching; multicellular organism reproduction; oogenesis; reproduction; body morphogenesis; apoptotic process

Cellular component: cytoplasm

clec-88 (K10B2.3)

Molecular function: carbohydrate binding

K10B2.2

Biological process: proteolysis

Molecular function: serine-type carboxypeptidase activity

lin-23 (K10B2.1)

Biological process: body morphogenesis; determination of adult lifespan; embryo development ending in birth or egg hatching; hermaphrodite genitalia development; locomotion; negative regulation of cell proliferation; nematode larval development; neuron projection morphogenesis; receptor-mediated endocytosis

Cellular component: cytoplasm; nucleus
Molecular function: protein binding; protein dimerization activity

F58F12.1
Biological process: ATP synthesis coupled proton transport; embryo development ending in birth or egg hatching; nematode larval development; reproduction
Cellular component: mitochondrion; proton-trasport ATP syntase complex, catalytic core F(1)
Molecular function: proton-transporting ATP synthase activity, rotational mechanism

F58F12.4
Unclassified

F58F12.2
Unclassified

F58F12.3
Unclassified

zig-10 (T25D10.2)
Unclassified

btb-2 (T25D10.5)
Molecular function: protein binding

T25D10.1
Unclassified

spp-11 (T25D10.3 – partial duplication)
Unclassified

2. Chromosome IV:

7D: 505,050..701,113
50D: 560,240..1,024,886

Overlapping region: 560,240-701,113 = 140,874 bp
30 protein-coding ORFs

efn-4 (F56A11.3)
Biological process: cell migration involved in gastrulation; embryo development ending in birth or egg hatching; morphogenesis of embryonic epithelium; regulation of cell adhesion; reproduction; tail tip morphogenesis
Cellular component: axon, membrane, neuronal cell body

F56A11.7
Unclassified

F56A11.5
Molecular function: catalytic activity; molybdenum ion binding; pyridoxal phosphate binding

gex-2 (F56A11.1)
Biological process: axon guidance; body morphogenesis; dendrite development; embryo development; embryo development ending in birth or egg hatching; hermaphrodite genitalia

development; locomotion; nematode larval development; oviposition
Cellular component: cell junction; cytoplasm

F56A11.6
Biological process: embryo development ending in birth or egg hatching

C18H7.12
Unclassified

C18H7.5
Unclassified

C18H7.6
Unclassified

C18H7.4
Biological process: protein phosphorylation
Molecular function: protein binding; protein kinase activity; protein tyrosine kinase activity

C18H7.7
Unclassified

C18H7.11
Unclassified

srt-59 (C18H7.8)
Unclassified

prmt-4 (C18H7.9)
Unclassified

col-102 (C18H7.3)
Molecular function: structural constituent of cuticle

inx-18 (C18H7.2)
Cellular component: gap junction

C18H7.1
Unclassified

nhr-76 (C05G6.2)
Biological process: regulation of transcription DNA-templated; steroid hormone mediated signaling pathway
Cellular component: nucleus
Molecular function: sequence-specific DNA binding; sequence-specific DNA binding transcription factor activity; steroid hormone receptor activity; zinc ion binding

K11H12.9
Biological process: protein phosphorylation
Molecular function: ATP binding; protein kinase activity

K11H12.1
Unclassified

rpl-15 (K11H12.2)
Biological process: apoptotic process; embryo development ending in birth or egg hatching; molting cycle, collagen and cuticulin-based cuticle; nematode larval development;

positive regulation of multicellular organism growth; reproduction; translation

Cellular component: ribosome

Molecular function: structural constituent of ribosome

K11H12.8

Unclassified

K11H12.7

Unclassified

K11H12.6

Unclassified

K11H12.11

Unclassified

K11H12.3

Biological process: reproduction

K11H12.4

Unclassified

K11H12.10

Unclassified

K11H12.5

Unclassified

cutl-28 (F41A4.1)

Biological process: blood coagulation; determination of adult lifespan; proteolysis

Cellular component: extracellular region

Molecular function: protein binding

cutl-26 (Y55F3C.7 - partial duplication)

Unclassified

3. Chromosome V:

7B: 19,505,848..20,101,145
16B*: 19,295,123..19,839,705
16D: 19,746,828..19,885,746
16E*: 19,295,580..19,840,162
50A*: 19,780,484..19,972,052
50B: 19,781,064..19,972,507
50C: 19,659,829..19,976,506
50D: 19,780,935 ..19,966,260
50E: 19,780,952..19,966,162
66C: 19,393,526..20,054,330
66E*: 19,295,300..19,839,882
C2*: 19,295,101..19,839,683

Overlapping region: 19,781,064-19,839,683 = 58,620 bp

11 protein-coding ORFs

fbxa-118 (M162.8 – partial duplication)

Molecular function: protein-binding

fbxa-194 (M162.11)

Molecular function: protein-binding

srt-45 (M162.3)

Unclassified

clec-258 (M162.2)

Molecular function: carbohydrate-binding

clec-259

Molecular function: carbohydrate-binding

M162.7

Unclassified

Y116F11B.2

Unclassified

daf-28 (Y116F11B.1)

Biological processes: dauer larval development; determination of adult lifespan; regulation of insulin receptor signaling pathway; regulation of transcription factor import into nucleus

Cellular components: extracellular regions; extracellular space

Molecular functions: hormone activity; insulin receptor binding

Y116F11B.17

Unclassified

pcp-4 (Y116F11B.3)

Biological processes: proteolysis

Cellular components: membrane raft

Molecular functions: serine-type peptidase activity

srw-38 (Y116F11B.5)

Cellular components: integral component of membranes

Overlapping Deletions:

4. Chromosome X:

16A: 817,573..830,086

19A: 800,773..827,100

19E: 821,499..829,454

C5: 823,167..827,286

Overlapping region: 823,167-827,100= 3,934 bp

1 protein-coding ORFs

daf-3 (F25E2.5 - partial deletion)

Biological process: dauer larval development; negative regulation of transcription from RNA polymerase II promoter; regulation of pharyngeal pumping; regulation of transcription, DNA-templated; transforming growth factor beta receptor signaling pathway

Cellular component: condensed chromosome; cytoplasm; intracellular; nucleus; transcription factor complex

Molecular function: enhancer sequence-specific DNA binding; sequence-specific DNA binding transcription factor activity

5. <u>Chromosome V:</u>

   16D: 7,663,133..7,687,447
   19C: 7,642,395..7,682,740
   50B: 7,650,284..7,693,435
   50C: 7,647,125..7,696,096
   50D*: 7,653,667..7,680,465
   50E*: 7,652,044..7,682,914

   Overlapping region: 7,663,133-7,680,465= 17,333 bp
   4 protein-coding ORFs
   - C12D5.5
     - Unclassified
   - C12D5.4
     - Unclassified
   - C12D5.3
     - Unclassified
   - *Cyp-33A1* (C12D5.7 - partial deletion)
     - Biological process: oxiation-reduction process
     - Molecular function: heme binding; iron ion binding; oxidoreductase activity, acting on donors, with incorporation or reduction of molecular oxygen

6. <u>Chromosome X:</u>

   66D: 7,528,608..7,529,729
   66E: 7,528,608..7,529,729
   C3*: 7,527,813..7,529,236

   Overlapping region: 7,528,608-7,529,236= 629 bp
   1 protein-coding ORFs
   - *ceh-14* (F46C8.5 - partial deletion)
     - Biological process: regulation of transcription, DNA-tempated; thermosensory behaviour
     - Cellular component: nucleus
     - Molecular function: DNA binding; protein binding; sequence-specific DNA binding; sequence-specific DNA binding transcription factor activity; zinc ion binding

7. <u>Chromosome I:</u>

   C1: 15,060,622..15,071,438
   C2: 15,060,388..15,071,427
   C4: 15,060,388..15,071,427
   C5: 15,061,973..15,071,438

   Overlapping region: 15,061,973..15,071,427= 9,455 bp
   4 rRNA genes
   - *rrn-1.1* (F31C3.7)
   - *rrn-2.1* (F31C3.11)
   - *rrn-3.1* (F31C3.9)

rrn-1.2 (F31C3.8)

8. Chromosome X:
    50C: 1,029..273,082
    50D: 1,029..295,671

    Overlapping region: 1,029..273,082 = 272,054 bp

    CE7X_3.1
        Unclassified
    Y73B3A.1
        Unclassified
    Y73B3A.20
        Unclassified
    Y73B3A.18
        Biological process: embryo development ending in birth or egg hatching, hermaphrodite genitalia development, reproduction
    Y73B3A.3
        Biological process: embryo development ending in birth or egg hatching
    Y73B3A.4
        Unclassified
    elk-2 (Y73B3A.5)
        Biological process: embryo development ending in birth or egg hatching, hermaphrodite genitalia development, negative regulation of vulval development
    fbxa-221 (Y73B3A.15)
        Molecular function: protein binding
    fbxa-222 (Y73B3A.22)
        Molecular function: protein binding
    fbxa-16 (Y73B3A.14)
        Unclassified
    Y73B3A.13
        Unclassified
    Y73B3A.7
        Unclassified
    cal-6 (Y73B3A.12)
        Biological process: embryo development ending in birth or egg hatching, receptor-mediated endocytosis, reproduction
        Molecular function: calcium-ion binding
    Y73B3A.8
        Unclassified
    Y73B3A.11
        Unclassified
    Y73B3A.9
        Unclassified
    Y73B3A.10

Biological process: cellular protein metabolic process, reproduction
Molecular function: ATP binding

T08D2.1
Biological process: locomotion, transport
Cellular component: integral component of membrane

T08D2.4
Molecular function: protein binding, zinc ion binding

T08D2.5
Unclassified

T08D2.6
Unclassified

T08D2.7
Biological process: protein phosphorylation
Molecular function: ATP binding, protein binding, protein kinase activity, transferase activity, transferring phosphorus-containing groups

T08D2.8
Molecular function: binding

Y73B3B.1
Molecular function: protein binding

Y73B3B.3
Unclassified

set-28 (Y73B3B.2)
Molecular function: protein binding

AC8.4
Unclassified

AC8.3
Unclassified

AC8.7
Unclassified

AC8.11
Unclassified

AC8.10
Unclassified

AC8.12
Unclassified

set-33 (Y108F1.3)
Biological process: embryo development ending in birth or egg hatching, nematode larval development, RNA interference
Molecular function: protein binding

math-43 (Y108F1.4)
Unclassified

Y108F1.5 (partial deletion)
Molecular function: helicase activity, protein binding
